# Long‐term cell fate and functional maintenance of human hepatocyte through stepwise culture configuration

**DOI:** 10.1096/fj.202201292RR

**Published:** 2023-01-06

**Authors:** Go Sugahara, Yuji Ishida, Jae Jin Lee, Meng Li, Yasuhito Tanaka, Hyungjin Eoh, Yusuke Higuchi, Takeshi Saito

**Affiliations:** ^1^ Department of Medicine, Division of Gastrointestinal and Liver Diseases University of Southern California, Keck School of Medicine Los Angeles California USA; ^2^ Research and Development Department PhoenixBio, Co., Ltd Hiroshima Japan; ^3^ Department of Molecular Microbiology & Immunology University of Southern California, Keck School of Medicine Los Angeles California USA; ^4^ Bioinformatics Service Program University of Southern California, Norris Medical Library Los Angeles California USA; ^5^ Department of Gastroenterology and Hepatology, Faculty of Life Sciences Kumamoto University Kumamoto Japan; ^6^ Department of Molecular Medicine Beckman Research Institute of City of Hope Duarte California USA; ^7^ USC Research Center for Liver Diseases Los Angeles California USA

**Keywords:** DMSO, DMSO2, human hepatocyte, humanized liver chimeric mice, primary human hepatocyte

## Abstract

Human hepatocyte culture system represents by far the most physiologically relevant model for our understanding of liver biology and diseases; however, its versatility has been limited due to the rapid and progressive loss of genuine characteristics, indicating the inadequacy of in vitro milieu for fate maintenance. This study, therefore, is designed to define environmental requirements necessary to sustain the homeostasis of terminally differentiated hepatocytes. Our study reveals that the supplementation of dimethyl sulfoxide (DMSO) is indispensable in mitigating fate deterioration and promoting adaptation to the in vitro environment, resulting in the restoration of tight cell–cell contact, cellular architecture, and polarity. The morphological recovery was overall accompanied by the restoration of hepatocyte marker gene expression, highlighting the interdependence between the cellular architecture and the maintenance of cell fate. However, beyond the recovery phase culture, DMSO supplementation is deemed detrimental due to the potent inhibitory effect on a multitude of hepatocyte functionalities while its withdrawal results in the loss of cell fate. In search of DMSO substitute, our screening of organic substances led to the identification of dimethyl sulfone (DMSO2), which supports the long‐term maintenance of proper morphology, marker gene expression, and hepatocytic functions. Moreover, hepatocytes maintained DMSO2 exhibited clinically relevant toxicity in response to prolonged exposure to xenobiotics as well as alcohol. These observations suggest that the stepwise culture configuration consisting of the consecutive supplementation of DMSO and DMSO2 confers the microenvironment essential for the fate and functional maintenance of terminally differentiated human hepatocytes.

**Abbreviations**
2D: two‐dimensional
ADH: alcohol dehydrogenase
ALDH: aldehyde dehydrogenase
ACN: acetonitrile
DEG: diethylene glycol
DEGDME: diethylene glycol dimethyl ether
DEO: 1,4‐dioxane
DMA: dimethylacetamide
DMF: dimethylformamide
DMS: dimethyl sulfide
DMSO: dimethyl sulfoxide
DMSO2: dimethyl sulfone
DMSOM: dimethylsulfoximine
HBV: hepatitis B virus
HH: Human hepatocytes
HLCM: humanized liver chimeric mice
HLCM‐HH: HLCM passaged human hepatocytes
IB: immunoblotting
IFA: immunofluorescent microscopic analysis
LC–MS: liquid chromatography‐mass spectrometry
NTCP: Na^+^‐Taurocholate Cotransporting Polypeptide
PHH: primary human hepatocytes
tBA: tert‐Butanol
TMSO: tetramethylene sulfoxide
UGTs: UDP‐glucuronosyltransferase

## 1 INTRODUCTION

Hepatocyte, the sole parenchymal cell of the liver, governs a broad range of essential biological processes for the maintenance of systemic homeostasis, including nutrition metabolism, storage, and distribution as well as detoxification of both endogenous and exogenous substances.^1^ Consequently, functional decline or a reduction in the number of hepatocytes leads to the manifestation of systemic pathological changes, the most advanced of which is liver failure, the 11th leading cause of death worldwide.^2^ To mitigate the burden of liver diseases, furthering our understanding of hepatocyte biology and pathophysiology plays a crucial role.

In this regard, the hepatocyte culture system is an essential platform in addressing the molecular basis of liver disorders. It also plays a fundamental role in drug development by supporting the assessment of pharmacokinetics and hepatotoxicity. While several types of in vitro study models have been established such as primary rodent hepatocytes and human hepatoma cell lines, each has distinct but critical constraints including interspecies differences and marginal relevance to the original primary cells, respectively.^3^, ^4^, ^5^, ^6^, ^7^ Human hepatocytes (HH), on the other hand, have been regarded as a research tool with limited versatility due to a rapid and progressive deterioration of the proper characteristics during in vitro culture.^8^ The decline in quality has been ascribed to a combination of the following factors; ischemic injury during organ procurement, mechanical stress resulting from collagenase perfusion for cell isolation, and cryoinjury associated with the freeze–thaw cycle for the preservation, and inadequacy of in vitro milieu required for the maintenance of genuine characteristics of HH.^8^, ^9^, ^10^

The optimization of hepatocyte culture medium is considered to play a key role in promoting the recovery from stresses/injuries associated with the cell procurement process and supporting the long‐term cell fate maintenance. The constituents of HH culture medium were initially adopted from the condition established for rodent hepatocytes^11^, ^12^; thereafter continuously evolving. Presently, the defined medium commonly utilized for the monolayer HH culture is supplemented with dexamethasone, insulin, epidermal growth factor, ascorbic acid, and dimethyl sulfoxide (DMSO).^13^, ^14^, ^15^ Of these, DMSO supplementation is considered to play a predominant role; however, it also has been shown to greatly impairs the activities of multiple enzymes involved in essential liver functions, including but not limited to alcohol dehydrogenase (ADH) and alkaline phosphatase.^16^, ^17^ Moreover, it potently induces the expression of multiple phase I and II drug‐metabolizing enzymes to supraphysiological levels while considerably inhibiting their enzymatic activities.^18^, ^19^, ^20^ Thus, the presence of DMSO in the culture medium is another major impediment to the versatility of HH.

To overcome these fundamental obstacles, herein, we established a novel two‐dimensional (2D) HH culture system, consisting of a stepwise culture approach. The initial phase is designed to mitigate the quality deterioration inflicted by cell procurement processes and foster the adaptation to in vitro environment. Our study determined that, despite undesirable effects on hepatocytic functions, DMSO supplementation is indispensable for cell fate restoration and maintenance. The completion of the initial phase culture with DMSO‐containing medium, namely recovery phase, results in the re‐establishment of a homeostatic state with features of terminally differentiated hepatocytes, including the polygonal morphology, polarization with bile canaliculi formation, and the expression of hepatocyte marker genes at levels comparable to those of normal liver tissue. However, the presence of DMSO is deemed undesirable beyond the recovery phase culture, since it potently inhibits multitude of hepatocyte functions. In search of DMSO substitute, our screening of miscible organic compounds led to the discovery of dimethyl sulfone (DMSO2), which minimizes the adverse effect of DMSO while exhibiting a comparable effect on cell fate maintenance.

Consequently, present study resulted in the development of a novel, stepwise culture configuration with DMSO‐DMSO2 supplementation, which confers the microenvironment required for the longevity and the functionality of terminally differentiated HH in vitro.

## 2 MATERIALS AND METHODS

### 2.1 Cells and tissue

Cryopreserved primary human hepatocytes (PHH) were obtained from either BioIVT (Westbury, NY, USA) or Corning (Corning, NY, USA). HH freshly isolated from the liver of humanized liver chimeric mice (HLCM) (HLCM‐HH) were isolated from cDNA‐uPA+/−/SCID harboring HH at the replacement index greater than 70% via conventional collagenase‐perfusion followed by filter‐based purification as described previously.^21^ Both PHH and HLCM‐HH were plated on type I collagen‐coated cell culture dish and incubated at 37°C 5% CO_2_ in DMEM containing 10% FBS, HEPES (20 mM), NaHCO_3_ (44 mM), L‐proline (15 μg/ml), bovine insulin (0.25 μg/ml), dexamethasone (50 nM), epidermal growth factor (5 ng/ml), 0.1 mM L‐ascorbic acid 2‐phosphate, and penicillin G (100 IU/ml), and streptomycin (100 μg/ml) as described previously.^22^ For 2D monolayer HH culture, unless specified in the figure legends, 2.13 × 10^5^ cells/cm^2^ represents the optimal cell seeding density.^22^ For 3D culture, HLCM‐HH seeded into 96‐well format Ultra‐Low Attachment Surface Plates at cell density of 40 000 cells/well (Corning, NY, USA) for the establishment of three‐dimensional spheroid culture. All commercially available media formulated for HH culture used in this study are summarized in Table S1 and applied to the culture of HLCM‐HH according to the manufacture's protocol. Normal human liver tissues were obtained from subjects who underwent curative hepatectomy for the removal of an isolated benign or metastatic liver tumor as described previously.^23^

### 2.2 Animal

cDNA‐uPA+/−/SCID (uPA+/wt: B6;129SvEv‐Plau, SCID: C.B‐17/Icr‐scid/scid Jcl) strain was used for the production of HLCM as described previously.^21^ HLCM utilized in this study was established with primary human hepatocytes obtained from two different donors as described previously.^22^ All animal work were performed in accordance with the NIH guideline under the protocol approved by the IACUC at the University of Southern California as well as PhoenixBio, Ltd.

### 2.3 Chemical and biochemical analyses

ADH activity was determined by the rate of NAD conversion to NADH in the presence and absence of ethanol using cell lysates of freshly isolated HLCM‐HH prepared with gentle sonication in 1 × PBS/1% triton X‐100 using UV spectrometry for the detection of absorbance change at 340 nm as described previously.^24^ The activities of aldehyde dehydrogenase (ALDH) and UDP‐glucuronosyltransferase (UGTs) activities in cell lysates of HLCM‐HH were determined by ALDH and UGT Activity Assay kit (Biovision, Milpitas, CA, USA), respectively, according to the manufacture's protocol. The assessment of CYPs activity (CYP1A2, CYP3A4, and CYP2C9) was determined using P450‐Glo™ Assays (Promega, Madison, WI, USA) using HLCM‐HH that had completed the recovery phase culture in the presence of 2% DMSO‐containing medium. CYP2E1 activity was assessed using Vivid™ CYP2E1 Blue Screening Kit (Thermo Scientific, Waltham, MA, USA). Immunonephelometry‐beased quantification of human albumin was carried out with BM6050 autoanalyzer (JEOL, Tokyo, Japan) using LX Reagent Seiken Alb II (Eisen Chemical, Tokyo, Japan). Acetate concentration was measured by Acetate Colorimetric Assay Kit (Biovision, Milpitas, CA, USA). DMSO and DMSO2 were quantified via LC–MS using Agilent Accurate Mass 6220 time of flight (TOF) coupled to an Agilent 1200 liquid chromatography system. In brief, DMSO, DMSO2, and extracted total metabolites were separated on a Cogent Diamond Hydride type C column (gradient 3). The mobile phase A consisted of ddH2O with 0.2% formic acid and phase B consisted of acetonitrile with 0.2% formic acid. Detected ions were deemed metabolites on the basis of unique accurate mass‐retention time identifiers for masses exhibiting the expected distribution of accompanying isotopologs. The LC–MS data were acquired using Agilent MassHunter Quanlitative analysis (B.07.00). All chemicals used in this study are summarized in Table S1. Chemical substances as cell culture medium supplements were used at the same concentration to DMSO (281 mM) or the highest concentration without signatures of cell toxicity.

### 2.4 Virus

Culture supernatant of Huh7 cells transfected with plasmid encoding subcloned clinical isolate of hepatitis B virus (HBV) (genotype A; accession number AB246337) was used to inoculate HLCM for the large‐scale propagation of infectious viral particle as described previously.^25^ The aliquoted serum of the HBV‐infected HLCM (7.9 × 10^9^ copies/ml) was stored at −80°C and used for in vitro challenge at 10 genome equivalent (GEq)/cell in the presence of 4% PEG8000 (Promega, Madison, WI, USA) for 24 h followed by the replacement of inoculum with fresh cell culture medium.

### 2.5 Protein analyses

Immunoblotting analyses (IB): cell lysate was prepared with Pro‐Prep protein extraction solution (iNtRON Biotechnology, Seongnam, South Korea) supplemented with Protease Inhibitor Cocktail (MedChemExpress, NJ, USA) were subjected to an SDS‐PAGE followed by transfer to PVDF membranes for the detection of indicated proteins. Antibodies and its corresponding dilution factors used in this study are summarized in Table S2.

### 2.6 Immunofluorescent microscopic analysis

Cells were plated on polymer coated chamber slide, μ‐Slide 8‐well (ibidi GmbH, Gräfelfing, Germany) and fixed with 10% notarized formalin (30 min), permeabilized with 0.02% Triton X‐100 for 15 min and blocked with 5% (mass/vol) BSA for 1 h. Immunofluorescence assays were performed using the primary antibodies listed in Table S2 for overnight, followed by incubation with Alexa Fluor 488‐conjugated goat anti‐mouse IgG (1:2000) (Jackson Immuno Research Lab, West Grove, PA, USA). Then, cells were subjected to nuclear staining and F‐actin staining using DAPI (Vector Labs. Inc., Burlingame, CA, USA) and Phalloidin (Biotium, Fremont, CA, USA), respectively. Microscopic images were captured with Leica TCS SP8 confocal microscopy system (Leica, Wetzlar, Germany) at the Cell and Tissue Imaging Core of the University of Southern California Research Center for Liver Diseases.

### 2.7 Nucleic acid

Cellular total RNA extraction was carried out using RNA Lysis Buffer or DNA/RNA Shield (Zymo Research, Irvine, CA, USA) were subjected to the using Quick‐RNA™ Microprep Kit (Zymo Research, Irvine, CA, USA). The extracted total cellular RNA was applied to cDNA synthesis with qScript cDNA SuperMix (Quantabio, Beverly, MA) for 2‐step RT‐qPCR analysis with Applied Biosystems™ PowerUp™ SYBR™ Green Master Mix (Applied Biosystems, Foster city, CA, USA), or subjected to a probe‐based 1‐step RT‐qPCR with TaqMan Fast Virus 1‐Step Master Mix for the detection of HBV genome. The primer and probe sequences are summarized in Table S3. Poly‐A‐based mRNA sequencing was carried out cDNA library synthesized with NEBNext Ultra™ II RNA Library Prep Kit for Illumina (New England BioLabs, UK) using Illumina NovaSeq 6000 Sequencing System.

### 2.8 Bioinformatics

RNA‐seq data were analyzed with Partek Flow version 4 (Partek Inc.). Raw sequencing reads were first trimmed from both ends with Quality Score method (bases with quality score less than 20 were trimmed from both ends, and trimmed reads shorter than 25 nt were excluded from downstream analyses). Trimmed reads were then mapped to the human genome hg38 using Star version 2.4.1d with default parameter settings and using Gencode v23 annotation as guidance.^26^ Gencode v23 annotation was used to quantify the aligned reads to genes/transcripts using Partek E/M method. Finally, read counts per gene/transcript in all samples were normalized using Upper Quartile normalization^26^ and analyzed for differential expression using Partek Gene Specific Analysis method (genes/transcripts with less than 10 reads in any sample among a data set were excluded). ANOVA FDR < 0.1, FC > 2 were employed for the differential expression analysis. RNA sequencing data are deposited at NCBI under accession code GSE 207912.

### 2.9 Statistics

Statistical analysis was performed using Prism version 5 (GraphPad). Significant differences were determined by Student's *t*‐test or one‐way ANOVA followed by Dunnett's test. Data are presented as means ± SD. *p* values of *p < .05, **p < .01, ***p < .001 determined by unpaired *t* test or ANOVA were considered significant. Displayed results were obtained from at least three independent experiments.

## 3 RESULTS

### 3.1 Essential role of seeding density and DMSO supplementation for HH fate recovery

Both cryopreserved and freshly isolated HH are subjected to variable degrees of cellular stress and damage during the cell procurement and preservation processes. Thus, we first evaluated the cell viability of HH, PHH and HH freshly isolated from the liver of HLCM‐HH, via Trypan blue assay, followed by the assessment of platability, defined as the rate of viable cells attachment to the culture dish.^1^ The result showed that the correlation between the viability and the platability of PHH is highly unpredictable as it varies greatly from donor‐to‐donor, which hinders our ability to precisely control the confluency (Figure S1A and Table S4). Moreover, the platabilities of PHH are overall lower than those of HLCM‐HH, which consistently exceed 60% (Figure S1A,B, and Table S4). The comparison of cryopreserved and freshly isolated HLCM‐HH from the same donor animals indicated the deleterious impact of cryopreservation and thawing process on cell viability, and more significantly, on platability (data not shown).

We next set to assess the role of cell seeding density on cell fate restoration as previous studies, including ours, have demonstrated the effect of culture confluency on hepatocellular functions.^22^, ^27^ Our assessment with HLCM‐HH reveals that a theoretical seeding density at 130% (~2.13 × 10^5^ cells/cm^2^), by taking the platability and median size of HH into account, permits cells to populate at full‐confluency on the culture dish (hereafter, referred to as optimal seeding density), which promote a formation of edge‐to‐edge monolayer adherence with tight cell–cell contact (Figure S1B,C). Moreover, with the optimal seeding density, HH regain the proper polarity, promoting the formation of bile canaliculi, as evidenced by the abundant expression of an apical domain bile canalicular transporter, MRP2, between plasma membrane (Figure S1C). Conversely, culture at less‐than‐optimal confluency leads HH to extend laterally, become flattened, and fail to promote the formation of bile canaliculi (Figure S1B,C).

**FIGURE 1:**
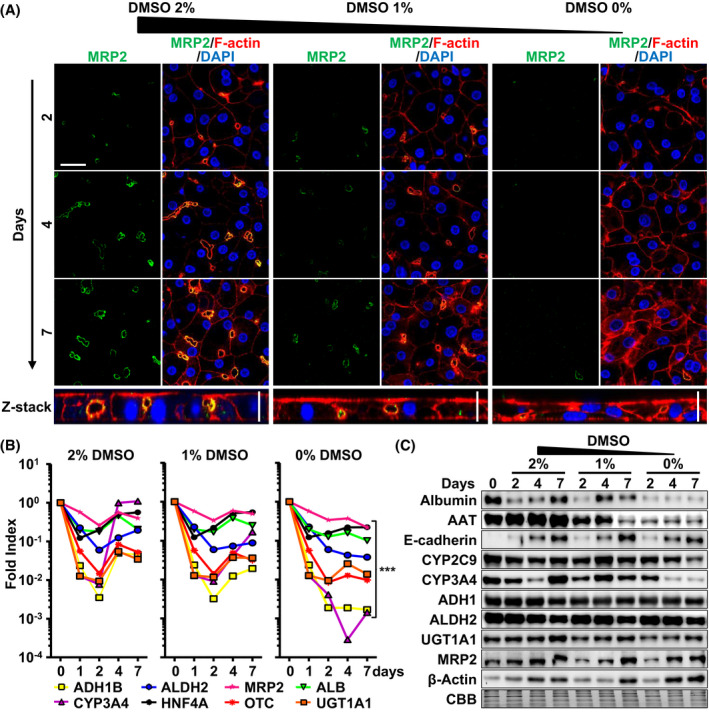
Impact of dimethyl sulfoxide (DMSO) on morphological and functional recovery of HLCM‐HHs. (A–C) HLCM‐HHs seeded at the optimal density were incubated with medium containing a decreasing concentration of DMSO for indicated duration followed by the immunofluorescent microscopic analysis (IFA) (A) for the detection of MRP2 and F‐actin and the side view analysis of the merged z‐stack image (day 7) in each condition. Green: MRP2, Red: Phalloidin (F‐Actin), and Blue: DAPI. Bar = 25 μm. Total cell lysates harvested at the specified time points were subjected to the assessment of indicated hepatocyte marker gene expression at the level of transcript (B) and protein (C) via RT‐qPCR and immunoblotting analysis (IB), respectively. The qPCR results represent the relative fold index to the average of the baseline (day 0) normalized by the value of GAPDH. For the IB analysis, an equal amount of protein samples from each condition were applied, with the CBB staining of the SDS‐PAGE gel serving as a loading control. *** indicate *p* < .001, versus 2% value at day 7 after plating by Student's *t*‐test.

Next, we assessed the role of cell culture media composition in preventing cell quality decline and promoting cell fate recovery. To this end, we first employed freshly isolated HLCM‐HH, which is superior to PHH in attaining the optimal cell density, and tested with medium devoid of each ingredient, such as dexamethasone, EGF, insulin, ascorbic acid, and DMSO, among which DMSO exhibit the predominant effect in alleviating cell quality deterioration and promoting cell fate recovery (Figure 1). HH cultured with DMSO (2% v/v (286 mM); hereafter 2% DMSO) undergo a dynamic morphological change, from oval shape to the polygonal appearance of matured mono‐ or binucleated HH by forming well‐established tight cell–cell contact along with bile canaliculi formation (Figure 1A). Conversely, HH cultured with decreasing concentration of DMSO resulted in the failure to restore of the morphology of genuine HH (Figure 1A). Without DMSO, despite being seeded at the optimized density, HH become flattened, overlying one another, which is dissimilar morphological aberration observed in cells plated at a less‐than‐optimal density (Figures 1A and S1C). The analysis of well‐accepted hepatocyte marker gene expression over the course of HLCM‐HH culture revealed a transient decline; however, they recovered to baseline levels within 4–7 days in the presence of 2% DMSO, whereas the culture with lower DMSO concentrations fail to restore the expression of these genes (Figure 1B,C). The criticalness of DMSO supplementation for the HH fate recovery was further confirmed with cryopreserved PHH that were seeded at the optimal cell density (Figure S1D). Furthermore, a series of commercially available media formulated for HH culture, which contains no or lower concentrations of DMSO, all failed to support cell fate restoration in marker gene expression, morphology and function (Figure S1E–H). These data collectively indicate that HH seeded at the optimal density requires up to 7 days to restore the cell fate, during which time the supplementation of 2% DMSO plays a pivotal role; hereafter, this timeframe will be referred to as the recovery phase culture. Notably, HH that have undergone 7 days of recovery phase culture demonstrate hepatocyte marker gene expression at comparable levels to those of HLCM liver tissue and normal human liver tissue (Figure S1I).

Lastly, we compared the expression abundance of hepatocyte marker genes expressed in HH that had completed the recovery phase with those in HH 3D spheroids. To this end, HLCM‐HH isolated from the same donor animals were seeded into ultra‐low attachment plates and cultured with the identical medium used for the restoration phase culture, resulting in the formation of spheroid with well‐defined perimeters (Figure S1J). We found that the HH culture with these two distinct methodologies, using the culture medium containing 2% DMSO, express essentially comparable level of hepatocyte marker genes (Figure S1K).

### 3.2 Criticalness of DMSO in the cell fate maintenance of HH

**FIGURE 2:**
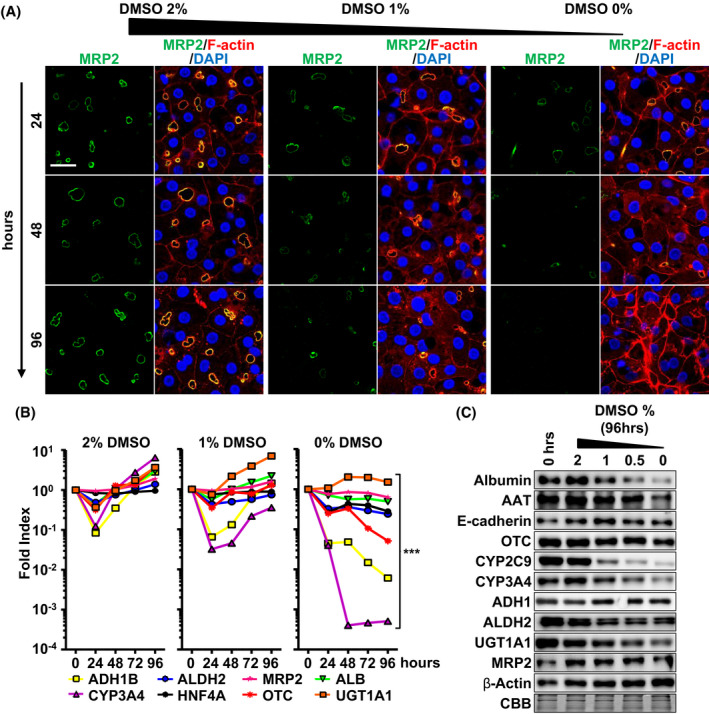
Impact of dimethyl sulfoxide (DMSO) on the cell fate maintenance and functionalities of HLCM‐HHs. (A–C) Fleshly isolated HLCM‐HHs cultured with 2% DMSO‐containing medium for 7 days were then incubated with a medium containing a decreasing concentration of DMSO for up to 96 h followed by IFA (A) for the detection of MRP2 and F‐actin. Green: MRP2, Red: Phalloidin (F‐actin), and Blue: DAPI. Bar = 25 μm. Total cell lysates harvested at the indicated time points were subjected to the assessment of indicated hepatocyte marker gene expression at the level of transcript (B) and protein (C) via RT‐qPCR and IB analysis, respectively. The qPCR results represent the relative fold index to the average of the baseline (0 h) normalized by the value of GAPDH. CBB staining serves as the loading control for IB analysis. *** indicate *p* < .001, versus 2% value after 96 h treatment after plating by Student's *t*‐test.

We next investigated the role of DMSO for the cell fate maintenance, for which HLCM‐HH that had completed the 7 days of recovery phase culture were incubated with medium containing decreasing concentration of DMSO (Figure 2). Our immunofluorescent microscopic analysis demonstrated that DMSO withdrawal led to the loss of the morphology characteristics of bona fide hepatocyte, such as polygonal cell shape, bile canaliculi, and actin filaments beneath the plasma membrane (Figure 2A). Indeed, we observed the complete loss of bile canaliculi, diffuse distribution of actin filament, and distorted cell shape by 96 h when HH are maintained in the absence of DMSO (Figure 2A). In parallel, we observed not only the decline in hepatocyte marker gene expression in a concentration and duration dependent manner (Figure 2B,C), but also increased expression of EMT‐ and hepatocyte progenitor/cholangiocyte‐marker genes in HH maintained with medium lacking DMSO (Figure S2). These findings suggest that DMSO remains an important cell culture medium constituent for the in vitro culture HH beyond the recovery phase to maintain the proper cell fate.

### 3.3 Adverse effects of DMSO that limit the versatility of HH

The primary biomedical applications of in vitro cultured HH are for the modeling of liver diseases as well as for the study of drug metabolism and hepatotoxicity, which often involves chronic rather than acute insults to hepatocytes; therefore, the capability to perform a stable, long‐term investigational treatment is warranted. Our observations thus far demonstrated that HH culture with medium containing a high concentration of DMSO (2%) promote the recovery and maintenance of the cell fate; however, its presence might hinder the utility of HH since it has been reported to profoundly dysregulate the function and/or abundance of multiple enzymes responsible for fundamental hepatic functions.^16^, ^17^, ^18^, ^19^, ^20^

**FIGURE 3:**
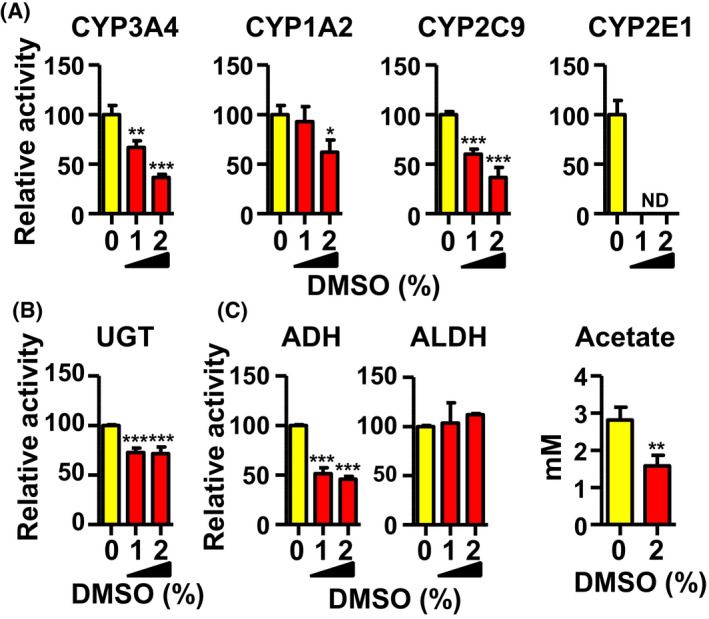
Inhibitory effects of dimethyl sulfoxide (DMSO) on metabolic functions of HHs. (A–C) Activities of phase I (CYP3A4, CYP1A2, CYP2C9, and CYP2E1) (A), phase II xenobiotics metabolizing enzymes (UGTs) (B), and ethanol metabolizing enzymes (ADH: left and ALDH: right) (C) in the presence of indicated DMSO concentrations were evaluated via luminescence, fluorescence, or colorimetrical absorbance‐based assays. For the assessment of ethanol metabolism to the biogenesis of acetate, HLCM‐HHs that had completed the recovery phase culture were treated with 5 mM ethanol for 24 h and the culture supernatant was subjected to quantification of acetate. Graph bars represent mean ± SD; *, ** and *** indicate *p* < .05 and *p* < .01*** and *p* < .001, versus 0% value by one‐way ANOVA; ND., not detected.

Thus, we evaluated the functionalities of HH maintained in DMSO‐containing medium, which revealed a significant impairment on the activities of multiple phase I drug metabolizing enzymes such as CYP3A4, CYP1A2, CYP2C9, and CYP2E1, in a concentration‐dependent manner, among which, the enzymatic activity of CYP2E1 was most strongly suppressed by DMSO (Figure 3A). These observations indicate that the inhibitory effect of DMSO spans a broad range of xenobiotics‐metabolizing enzymes, and the degree of inhibition is highly variable depending on the enzymes. In addition, we observed that DMSO induces the expression of CYP3A4 and CYP2E1, to an extent that further surpasses the physiological level, particularly upon the long‐term maintenance beyond the recovery phase culture (Figure S3).

We also tested whether DMSO impairs other aspects of hepatocyte function. Our assessment demonstrated that DMSO exerts a potent inhibitory effect on one of the phase II xenobiotics metabolism enzymes, UGTs (Figure 3B). Moreover, we found that DMSO substantially impair the capacity of alcohol metabolism at the level of ADH as well as CYP2E1, but not at the level of ALDH; thereby, acetate biogenesis from ethanol is prohibited (Figure 3C). These findings suggest that, even after the recovery phase culture is completed, the HH would be of limited utility as long as it is maintained in DMSO‐containing medium due to the significant functional impairments.

### 3.4 Identification of DMSO substitute that maintains the functionality of HH

**FIGURE 4:**
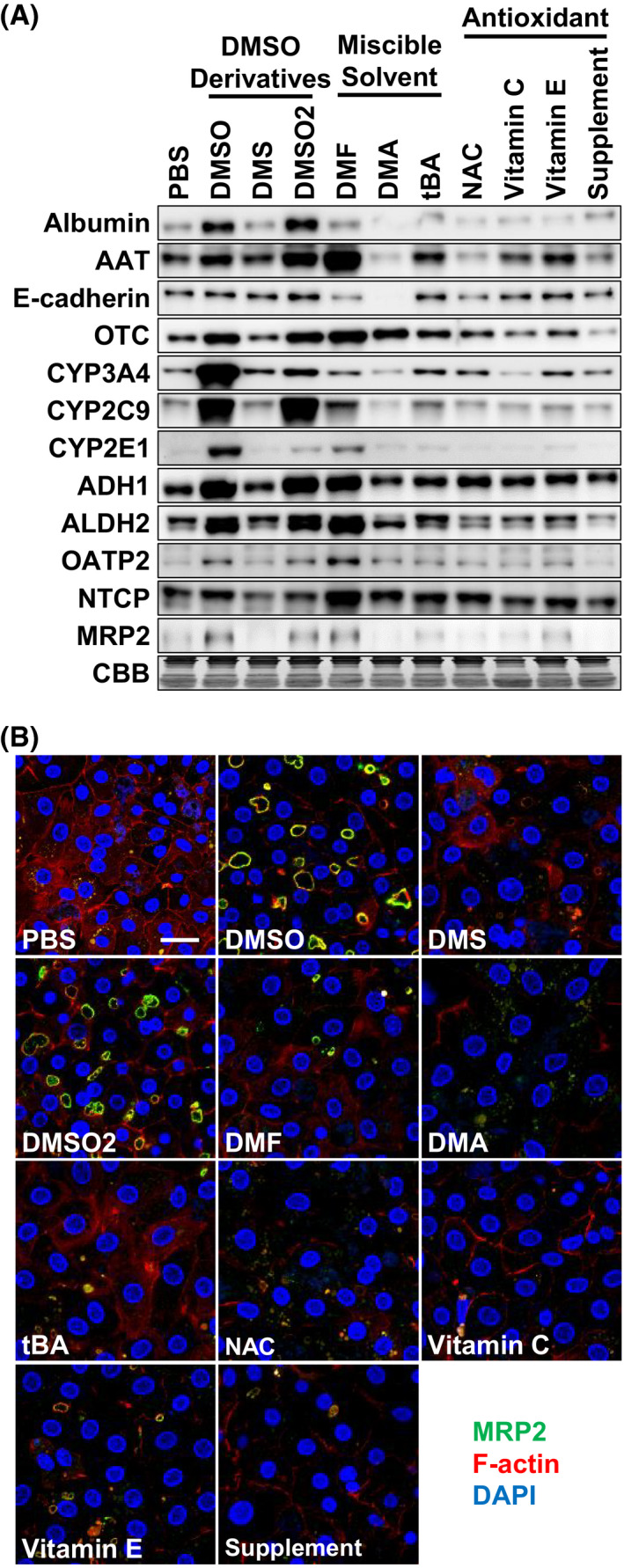
Effect of organic compounds and antioxidant reagents on the cell fate maintenance and function of HLCM‐HHs. (A and B) Freshly isolated HLCM‐HHs were cultured with 2% DMSO containing medium for 7 days followed by incubation with culture medium containing indicated substances at the concentration described in Table S1 for an additional 7 days for the morphological assessment via IB (A) and IFA (B) analysis of hepatocyte marker gene expression. For the IB analysis, an equal amount of protein samples from each condition were applied, with the CBB staining of the SDS‐PAGE gel serving as a loading control. The IFA image represents merged image of MRP2 (green), Phalloidin (F‐actin) (red) and DAPI (blue). Bar = 25 μm.

The effect of DMSO on the modulation of cellular biochemistry spans from the redox potential, molecular interaction, protein folding, DNA structure, to lipid membrane integrity.^28^, ^29^, ^30^, ^31^, ^32^ These biochemical modulatory properties are believed to act in cooperation to regulate a broad range of cell biology processes, including but not limited to the cell cycle, differentiation, programmed cell death, polarization, lipid metabolism, glycosylation, and autophagy.^30^, ^33^ Of those, the modulation of cellular redox state is one of the most well‐accepted cell biological effects of DMSO. Thus, we first examined the effect of either reduced or oxidized forms of DMSO, dimethyl sulfide (DMS) and DMSO2, respectively, on the maintenance of hepatocyte characteristics. The cell culture medium containing DMSO2, but not DMS, exhibited a comparable effect to that of DMSO for the maintenance of HH morphology, as well as the expression of marker genes at the levels of both transcript and protein (Figures 4A,B and S4A).

In human body, DMSO could be either oxidized to DMSO2 or reduced to DMS,^34^, ^35^, ^36^ respectively; thereby, it is conceivable that the effect of DMSO and DMSO2 on the cell fate maintenance might result from the oxidative metabolism of DMSO or reduction of DMSO2 in HH. To address these possibilities, we first measured DMSO2 concentration in the culture supernatant over the course of HH incubation with DMSO‐containing medium via HPLC–MS approach (Figure S4B). While DMSO2 was detected at later time points, however, it merely reached a concentration that has a favorable impact on HH cell fate maintenance. Moreover, a comparable level of DMSO2 was detected upon incubation in the absence of cells (Figure S4B), indicating the contribution of atmospheric oxidation rather than the biogenesis in HH. Similarly, we observed that the HH culture with DMSO2‐containing medium does not promote the conversion to DMSO (Figure S4B). These observations suggest that the modulation of cellular redox state is not the predominant attribute for the favorable effect of DMSO and DMSO2 on the cell fate maintenance of HH. This notion is further affirmed by our observation in which a series of potent antioxidants showed a negligible effect on the maintenance of hepatocyte characteristics (Figures 4A,B and S4A). Our finding also suggests that these compounds do not function as the active metabolites of each other.

DMSO and DMSO2, which are both equipped with hydrophilic and amphiphilic property, may contribute to cell fate maintenance by altering a broad range of cellular biochemistry. Indeed, the regulatory effect of other water‐miscible organic compounds that are less active in redox modulation, such as dimethylacetamide (DMA), dimethylformamide (DMF), and tert‐Butanol(tBA), on cellular differentiation has been reported in studies with cancer cell lines.^37^, ^38^, ^39^, ^40^ Among those, we observed that DMF and tBA support the maintenance of some, but not all, hepatocyte marker gene expression either at transcript or protein level to a certain extent; however, their effects appear much inferior to those of DMSO and DMSO2 as none of these compounds supported the maintenance of proper morphological appearance (Figures 4A,B and S4A). These findings indicate that the effect of organic substances on HH cell fate maintenance is highly diverse, with differential effects on morphology and marker gene expression; thereby, a multifaceted assessment is required, as excessive reliance on a single readout result in the misinterpretation of their biological effects.

### 3.5 Extended screening of miscible chemicals for their effect on HH fate maintenance

**FIGURE 5:**
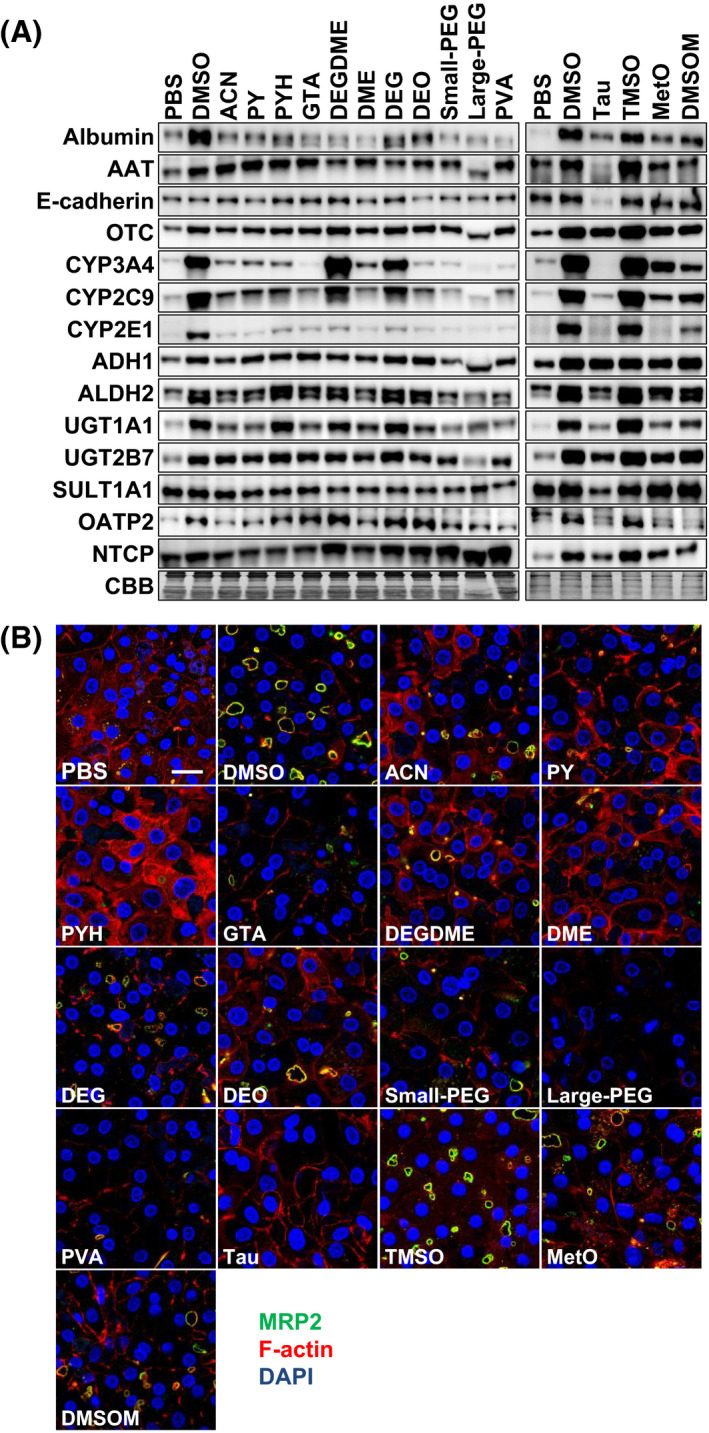
Extended screening of miscible compounds for their effect on hepatocyte characteristics. (A and B) Freshly isolated HLCM‐HHs were cultured with 2% DMSO containing media for 7 days, followed by incubation with culture medium containing the indicated compounds at the concentration described in Table S1 for an additional 7 days for morphological assessment through IB (A) and IFA (B) analysis of hepatocyte marker gene expression. For the IB analysis, an equal amount of protein samples from each condition were applied, with the CBB staining of the SDS‐PAGE gel serving as a loading control. IFA image represents merged image of MRP2 (green), Phalloidin (F‐actin) (red) and DAPI (blue). Bar = 25 μm.

To further elucidate the chemical properties involved in the cell fate maintenance of HH, we extended the screening of water‐miscible organic substances (Figure 5). These compounds were chosen for their suitability as a supplement to cell culture media in terms of stability, volatility, chemical safety and commercial availability, as well as similarities to DMSO and DMSO2, such as the presence of an oxidized sulfur atom. We also included water‐miscible mid‐ and macro‐size molecules, PEG 400 (Small PEG), PEG 20000 (Large PEG), and poly vinyl alcohol (PVA), which have low cell membrane permeability, thereby allowing us to assess the role of extracellular organic substances on HH cell fate maintenance. We observed that tetramethylene sulfoxide (TMSO), and to a much lesser extent, diethylene glycol (DEG), dimethylsulfoxonium methylide (DMSOM), and 1,4‐dioxane (DEO) have a favorable influence on hepatocyte marker gene expression while others had a modest, or perhaps negligible impact (Figures 5A and S5). We also found that certain compounds, in particular TMSO, have a positive impact on the preservation of cell architecture and polarity, as evidenced by the presence of bile canaliculi (Figure 5B).

### 3.6 Impact of DMSO alternatives on hepatic function

**FIGURE 6:**
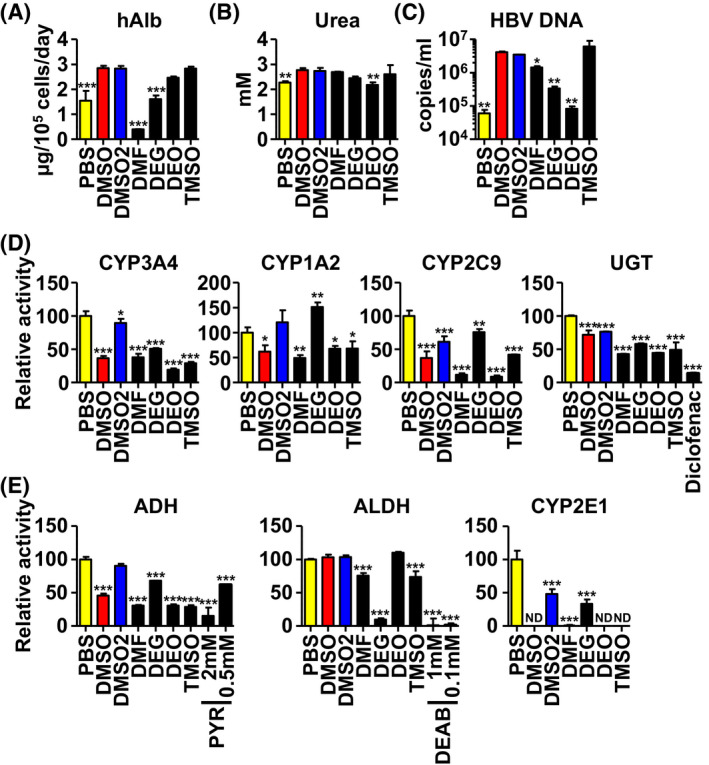
Impact of dimethyl sulfoxide (DMSO) substitute candidates on hepatocyte cell fate maintenance and their influence on hepatic metabolic activity. (A–C) Freshly isolated HLCM‐HHs that had completed the 7 days of recovery phase culture with 2% DMSO‐containing medium were incubated for an additional 7 days in the presence of indicated organic compounds followed by the assessment of hepatocyte functions on day 14: the secretion of albumin (A) and urea (B) into the culture medium, as well as HBV infection efficiency (C). HBV inoculation (MOI 10) was carried out at the end of the recovery phase culture on day 7. Graph bars represent mean ± SD; *, ** and *** indicate *p* < .05 and *p* < .01*** and *p* < .001, versus DMSO value by one‐way ANOVA). (D and E) Effect of indicated organic compounds on the activities of xenobiotics metabolizing enzymes (D) and ethanol metabolizing enzymes (E). CYP3A4, 1A2, and 2C9 activities in HLCM‐HHs that had completed the recovery phase culture were assessed via luminescence‐based assays in the presence of indicated organic compound. ADH, ALD and UGT activities in cell lysate of HLCM‐HH that had completed recovery phase culture were determined using colorimetrical absorbance‐based assays in the presence of indicated organic compound. CYP2E1 activity was determined via luminescence‐based assay using recombinant enzyme in the presence of indicated organic compound. Graph bars represent mean ± SD; *, ** and *** indicate *p* < .05 and *p* < .01*** and *p* < .001, versus Control (1xDPBS) value by one‐way ANOVA).

While restoring the proper cell morphology and a physiological level of marker gene expression are both critical prerequisites, the ultimate requirement for in vitro cultured HH is to demonstrate the maintenance of genuine hepatocyte function to serve as an experimental platform for the study of hepatic biology and diseases. To this end, HH that had completed the 7 days of recovery phase culture were maintained with medium supplemented with the potential DMSO substitutes, DMSO2, DMF, DEO, DEG, and TMSO, for 7 days, which were then used for the assessment of hepatic functions such as albumin secretion, susceptibility to HBV infection, urea production (Figure 6A–C).

In accordance with the detrimental effect of DMSO withdrawal (Figure 2), the albumin concentration in the culture supernatant of HH maintained with medium devoid of organic substances was significantly lower than that of HH maintained with DMSO (Figure 6A). On the other hand, the culture supernatant of HH maintained with DMSO alternatives had comparably high levels of human albumin, with the exception of DMF and DEG, which were significantly lower compared to others. Next, we tested the effect of these compounds on urea cycle activity, which demonstrated an overall similar capacity except in HH maintained with DEO (Figure 6B).

We also evaluated the impact of these compounds on the susceptibility to HBV infection. HBV entry to hepatocyte requires the interaction between the viral envelope protein and Na^+^‐Taurocholate Cotransporting Polypeptide (NTCP), the host protein abundantly expressed on the cell surface of hepatocytes. Of important note, N‐glycosylation is essential for proper localization of NTCP at the plasma membrane, and DMSO supplementation has been demonstrated to play a significant role in modulating protein glycosylation machinery, including that of NTCP.^41^, ^42^, ^43^ Our findings show that the efficiency of HBV replication in HH maintained with DMSO2 or TMSO is equivalent to that with DMSO; however, HH cultured with all other substances, particularly DEO and DEG, failed to support robust HBV lifecycle (Figure 6C).

Next, we investigated the effect of these compounds on enzymes involved in phase I and II xenobiotics metabolism using HH that had completed the recovery phase culture and found a varying degree of inhibitory effect (Figure 6D). For example, all compounds tested significantly suppressed the enzymatic activity of CYP3A4, yet DMSO2 exhibited a considerably less inhibitory effect compared to others. CYP1A2 activity was inhibited by DMSO, DMF, DEO, and TMSO while DMSO2 and DEG showed no inhibitory effects. CYP2C9 and UGTs activities were significantly suppressed with all compounds tested, of those, DMF and DEO exhibited much greater degree of inhibitory effect compared with others.

Lastly, we tested the impact of DMSO alternatives on enzymes involved in alcohol metabolism such as ADH, ALDH, and CYP2E1 (Figure 6E) using either HH that had completed the 7 days of recovery phase culture or recombinant protein. In addition to the inhibitory effect of DMSO on ADH activity, we observed that almost all compounds, with the exception of DMSO2, demonstrated their potent antagonistic effect at a level equivalent to that of a well‐accepted ADH inhibitor, pyrazole. Their impact on the enzymatic activity of ALDH, on the other hand, was generally well preserved, except in the presence of DEG, which exerted a substantial inhibitory effect at a level equivalent to the ALDH inhibitor, DEAB. We also discovered that, other than DMSO2 and DEG, these substances abolish the function of CYP2E1, an alternative pathway in the oxidative metabolism of alcohol.

These observations collectively indicate that DMSO2 is the most desirable DMSO substitute that support both cell fate maintenance and functional preservation of in vitro cultured HH. Lastly, this notion holds true not only for HLCM‐HH, since the abundance of hepatocyte marker gene expression appears overall comparable in PHH that had been maintained with DMSO2‐ and DMSO‐supplemented medium (Figure S6).

### 3.7 Transcriptome analysis of HH maintained in DMSO2‐containing medium

To further characterize the effect of DMSO2 on HH characteristics, we conducted a transcriptome analysis and compared with that of HH maintained with DMSO or in the absence of any organic compounds. We also included hepatoma cell lines, HepG2 and Huh7 cells, which are commonly utilized as surrogates of HH, in the analysis.

**FIGURE 7:**
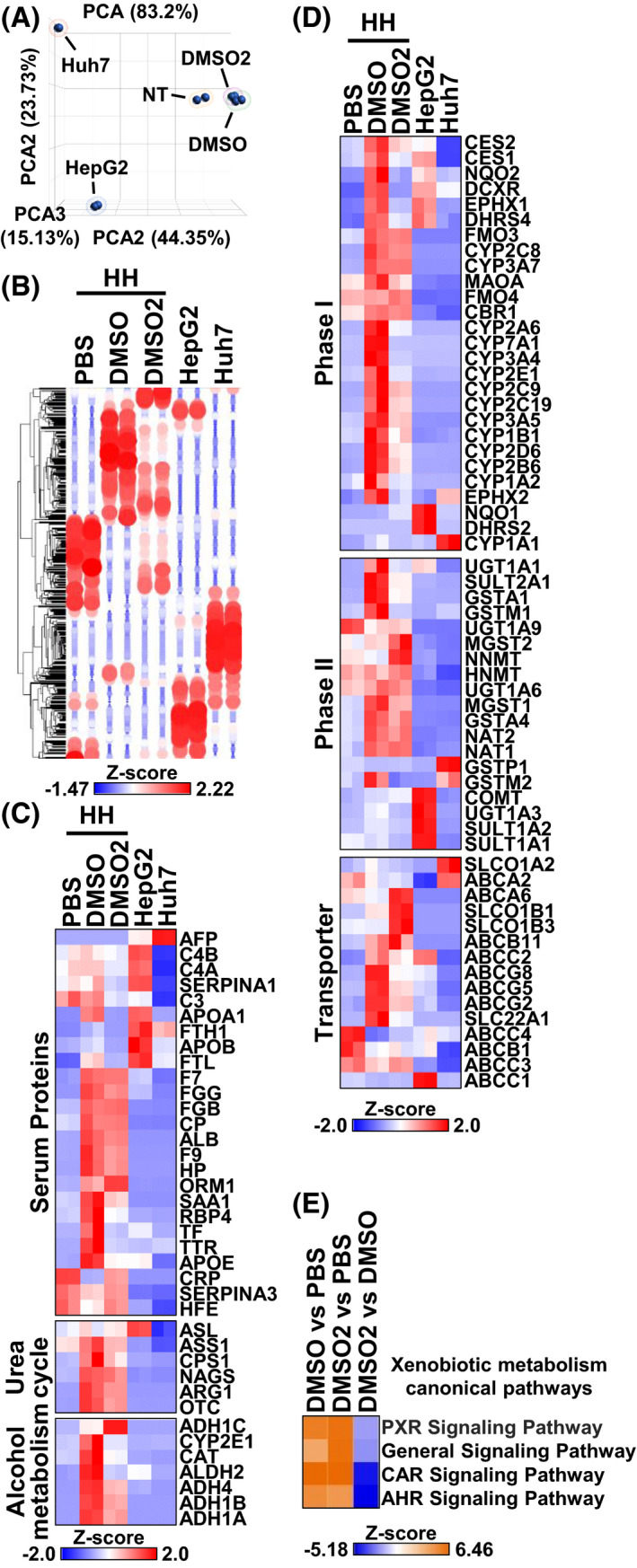
Shared and differential effects of dimethyl sulfoxide (DMSO) and DMSO2 on HH characteristics: transcriptome analyses. (A–E) Freshly isolated HLCM‐HHs that had completed the 7 days of the recovery phase culture in the presence of 2% DMSO containing medium were further maintained with either DMSO or DMSO2‐containing medium for an additional 7 days, which were then subjected to mRNA‐sequencing analysis. HepG2 cells and Huh7 cells cultured with 2% DMSO containing medium for 7 days were included in the analysis as comparison groups. Principal component analysis of normalized RNA‐seq data (A). Hierarchical clustering analysis of the differentially regulated genes (DEGs) (cutoffs used were FDR of 0.1 and a fold change of 2) (B). Comparison of normalized transcriptome data for genes involved in the indicated hepatocyte functionality (C and D). IPA canonical pathway analysis of DEGs for xenobiotic metabolism pathway (E).

The principal component analysis revealed, as expected, a stark contract between HH and hepatoma cell lines as well as a strong resemblance of transcriptome profiles between HH maintained with DMSO and DMSO2 (Figure 7A). Accordingly, the hierarchical clustering analysis illustrated the overall similarity between HH cultured with DMSO2 and DMSO while HH cultured without any organic compounds as well as hepatoma cell lines demonstrated highly distinctive transcriptome landscapes (Figure 7B). Moreover, our functional analysis revealed that genes responsible for serum proteins, urea cycle, and alcohol metabolism are overall well maintained in HH cultured with DMSO2 at a level comparable to that with DMSO (Figure 7C). These findings affirm our hypothesis that DMSO and DMSO2 both support the cell fate maintenance of terminally differentiated HH.

Nevertheless, our functional analysis also revealed that the overall expression abundance of drug‐metabolizing enzymes, in particular for phase I genes, is much greater in HH maintained with DMSO‐containing medium than that with DMSO2‐containing medium (Figure 7D). This finding is in accordance with the results of the pathway analysis, demonstrating the pronounced upregulation xenobiotics metabolizing pathways such as but not limited to PXR and CAR signaling pathways in HH maintained with DMSO‐containing medium (Figure 7E). Of important note, phase I enzymes highly upregulated in HH maintained with DMSO, such as but not limited to CYP3A4, CYP2C9, and CYP2E1, are the enzymes potently inhibited by DMSO (Figures 6D,E, 7D, and S3), suggesting that DMSO has dual regulatory properties as an inducer and inhibitor on numerous xenobiotic metabolizing enzymes.

Lastly, we assessed whether HH cultured in the presence of DMSO or DMSO2 skew their characteristics towards the periportal (zone I) and the perivenular hepatocytes (zone III) (Figure S7A,B). Our analysis suggests that the transcriptome profiles of HH maintained with DMSO or DMSO2 are unrelated to the gene expression patterns associated with hepatic zonation.

### 3.8 The versatility of HH maintained with DMSO2: The clinical implications

Our finding together indicates that the maintenance of HH with DMSO‐containing medium beyond the recovery phase results in the induction of drug‐metabolizing enzymes, such as CYP3A4 and CYP2E1, to supraphysiological levels meanwhile their enzymatic activities are substantially prohibited (Figures 6E and S3). Hence, the application of HH that had completed recovery phase culture for studies of drug‐metabolism and hepatotoxicity requires extra precaution since organic compounds, such as DMSO, present in the culture medium might exert inductive and/or inhibitory effect on enzymes involved in the metabolism or the adverse effect of testing drugs.

**FIGURE 8:**
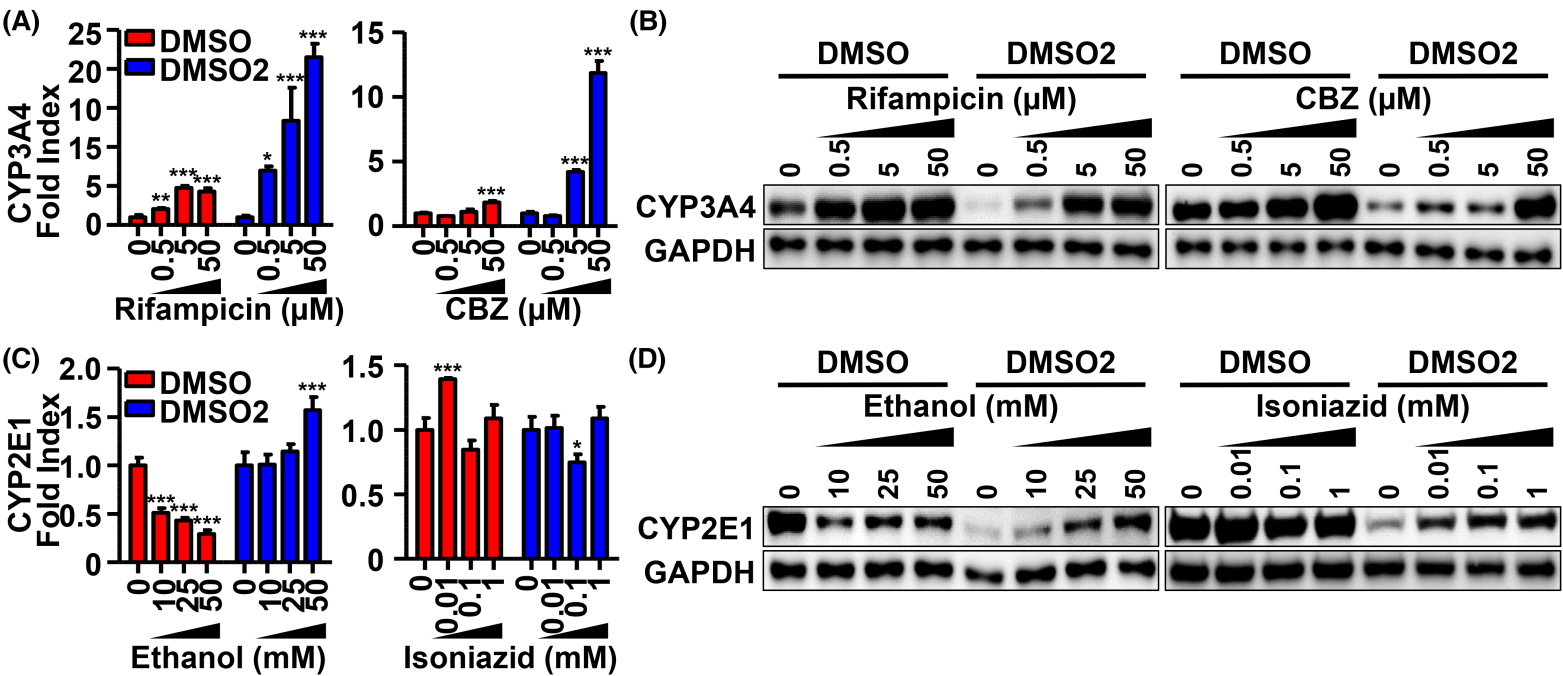
Inducibility of CYP genes in HLCM‐HHs maintained in culture medium containing dimethyl sulfoxide (DMSO) or DMSO2. (A–D) Freshly isolated HLCM‐HHs that had completed 7 days of the recovery phase culture in the presence of 2% DMSO containing medium were further maintained with either DMSO or DMSO2‐containing medium for an additional 7 days followed by treatment with indicated xenobiotics. Then HLCM‐HHs were subjected to the assessment of expression abundance of CYP3A4 (A and B) and CYP2E1 (C and D) at the level of transcript (A and C) and protein (B and D). Graph bars represent mean ± SD; *, ** and *** indicate *p* < .05, *p* < .01, and *p* < .001, versus control (without reagents) value in each condition by one‐way ANOVA.

To investigate the clinical implications of this scenario, HH that had completed the recovery phase culture were incubated with rifampicin or carbamazepine, two medications that are known to be potent inducers of CYP3A4, in the presence of either DMSO or DMOS2. We observed a broader dynamic range of CYP3A4 induction by these medications in HH cultured with DMSO2‐containing medium, especially at the subtherapeutic and therapeutic concentrations (Figure 8A,B). Similarly, we tested CYP2E1 inducibility by ethanol and isoniazid, both of which are known to upregulate CYP2E1 expression. These treatments led to the dose‐dependent upregulation of CYP2E1 in HH maintained in DMSO2‐containing medium, and this phenomenon was not observed in HH cultured with DMSO (Figure 8C,D).

## 4 DISCUSSION

HH in vitro culture system has been widely regarded as a research tool with limited utility due to the rapid loss of hepatocyte characteristics, which prompted the development of highly sophisticated culture systems, such as with 3D‐organoid technology and microscale culture with bioprinting technology; however, these approaches add an extra layer of technical complexity.^5^, ^44^, ^45^, ^46^, ^47^ With the ultimate goal of improving the versatility of HH, in this study, we developed a novel 2D HH culture system with stepwise culture configuration.

Our study revealed that seeding at a high confluence, which requires accurate assessment of the platability, permits HH to populate in close proximity to neighboring cells without compromising the characteristic polygonal shape, consequently promoting the reestablishment of proper cellular polarity. We also found that incubation with a high concentration of 2% DMSO is another requirement for the recovery of HH from stresses and injuries associated with the cell procurement procedure. These two critical prerequisites are not mutually exclusive; indeed, the HH culture with 2% DMSO alone is insufficient as cells seeded at a suboptimal confluency fail to restore the morphological appearance of matured hepatocytes as well as the expression of marker genes. Similarly, HH seeded at optimal density would not regain the characteristics of matured hepatocytes unless cultured with DMSO‐containing medium. Taken together, the concomitance of 2% DMSO and the optimal cell seeding density are both required for cell fate recovery as well as for prevention of dedifferentiation and/or trans differentiation.

Of important note, the effects of DMSO on promoting and maintaining the cellular differentiation are not limited to hepatocytes, but also neutrophils, thyroid cells, osteoblasts, neuronal cells, and erythroblasts.^48^, ^49^, ^50^, ^51^, ^52^, ^53^, ^54^, ^55^ To date, the mechanism by which DMSO supports the cell fate maintenance across different cell types remains entirely elusive. This is due in large part to the multifaceted effect of DMSO on cellular biochemistry, ranging from the modulation of redox potential, protein folding, DNA structure, molecular interaction, and lipid membrane integrity,^28^, ^29^, ^30^, ^31^, ^32^ which are expected to cooperatively alters a broad spectrum cell biology including, but not limited to, differentiation, cell cycle, programmed cell death, polarization, lipid metabolism, protein glycosylation, and autophagy.^30^, ^33^

Our work demonstrated that HH completed the 7 days of recovery phase culture could maintain the state of terminal differentiation for at least 21 days, if not longer, in the presence of 2% DMSO. Notably, the characteristics of HH that had completed the recovery phase culture well resemble those of hepatocytes within the normal liver tissue in terms of the abundance of hepatocyte marker gene expression as well as the morphological architectures. Moreover, the quality of HH, upon the completion of the recovery phase culture, appears comparable to that of HH spheroid. This observation contradicts the general perception that the monolayer culture are inferior to 3D culture systems, for which we postulate that the preservation of proper cellular architecture and polarity in our 2D system is the foremost critical attribute.

Our study indicates that HH that had completed the recovery phase culture may have a limited versatility as an experimental platform, particularly when maintained with DMSO‐containing medium owing to its significant inhibitory effect on multiple enzymes that are required for essential liver functions. Moreover, there might exist a reciprocal relationship, albeit not universal, between the degree of enzymatic activity inhibition and their expression induction by DMSO. For example, HH maintained in the presence of DMSO beyond the recovery phase results in the induction of CYP3A4, the phase I enzyme responsible for the metabolism of more than 50% of clinically relevant drugs, to a supraphysiological level meanwhile potently suppressing its enzymatic activity. Similarly, the expression abundance and enzymatic activity of CYP2E1 are both highly dysregulated in the presence of DMSO. These adverse effects of DMSO are expected to greatly limit the versatility of HH, particularly for the studies of drug metabolism, toxicology, and pharmacokinetics.

The application of HH for the modeling of liver disorders such as metabolic liver diseases also requires precaution if maintained in a medium containing DMSO. As our work demonstrated, DMSO significantly inhibits the activity of multiple enzymes involved in alcohol metabolism, such as ADH and CYP2E1, thereby limiting its utility for the studies of alcoholic liver diseases. Moreover, studies by others have reported that DMSO potently inhibits the enzymatic activities of aldose reductase, and peroxidase,^56^, ^57^ enzymes facilitating sugar metabolism and modulating redox state, respectively, implying that HH cultured with DMSO unlikely serve as an experimental platform for the studies of non‐alcoholic fatty liver diseases given the involvement of these enzymes in the pathophysiology.^58^, ^59^ These observations collectively emphasize that the restoration and maintenance of cell fate is insufficient to warrant the preservation of hepatocyte functions.

The substantial adverse effect on multiple hepatic functions points to the necessity of substituting DMSO with another substance at the completion of the recovery phase culture, especially when applying HH for research activities as it might confound the interpretation of study results. The requirements for DMSO alternatives are the following: (1) sufficiently support cell fate maintenance and (2) have insignificant or no off‐target effect on hepatocyte functions. In search of DMSO alternatives, our screening of miscible organic compounds resulted in the identification of a few candidates, among which DMSO2 offers the most favorable properties. Previous studies with other cell types proposed DMF and DMA as potential DMSO substitutes^38^, ^39^, ^40^; however, our assessment indicates these compounds are much inferior to DMSO2, at least for 2D culture of HH. We discovered that the effect of DMSO2 on the maintenance of hepatocyte morphology and marker gene expression is comparable to that of DMSO, whereas it outperforms DMSO in terms of the preservation of hepatocyte functions. In fact, HH cultured in the presence of DMSO2 maintain the expression abundance of xenobiotics‐metabolizing enzymes and their corresponding enzymatic activities at a level closer to the physiological conditions. The modest, perhaps insignificant, inhibitory effect on enzymes involved in the metabolism of alcohol is another example that supports the superiority of DMSO2 as DMSO substitute.

Lastly, our work also points to the necessity to refine surrogate markers of mature hepatocytes. For example, the expression abundance of CYPs and MRP2 is often employed as makers of terminally differentiated hepatocytes^60^; however, it is evident, based on our observations, that these genes could be artificially induced by certain chemical compounds supplemented to the cell culture medium, such as DMSO, DEGDME, DEG, and TMSO, leading to the potential overrepresentation of these markers. Similarly, despite the fact that the proper morphological appearance is one of the key quality indicators, it may not be sufficient to ascertain the fate and functionality of in vitro‐culture HH, as seen in HH maintained with DMSO and TMSO. Consequently, our study employed a multi‐readout strategy that consisted of the evaluation of marker gene expression, the microscopic analysis of cellular architecture and polarity, and the evaluation of liver functions, with conclusions based on the overall results of these multidimensional evaluations.

In conclusion, the stepwise supplementation of DMSO‐DMSO2 to the culture medium facilitates the comprehensive restoration and maintenance of human hepatocyte terminally differentiation along with well‐preserved hepatic functions, thereby greatly enhancing the versatility of in vitro cultured HH.

## AUTHOR CONTRIBUTIONS

Study concept and design: Takeshi Saito; in vitro experiment performance: Go Sugahara, Yuji Ishida; human hepatocytes preparation: Go Sugahara, Yuji Ishida; LC–MS analysis: Hyungjin Eoh, Jae Jin Lee, technical or material contribution: Yusuke Higuchi, Yasuhito Tanaka; data analysis and interpretation of data: Go Sugahara, Yuji Ishida, Meng Li, Takeshi Saito; drafting of the manuscript: Go Sugahara, Takeshi Saito; critical revision of the manuscript: Takeshi Saito.

## DISCLOSURES

The authors have declared no conflict of interest.

## Supporting information


Figure S1.

Figure S2.

Figure S3.

Figure S4.

Figure S5.

Figure S6.

Figure S7.

Table S1.

Table S2.

Table S3.

Table S4.

**Appendix S1.** Supporting Information.

## Data Availability

The data that support the findings of this study are available in the methods and results, for additional data are available on request from the corresponding author.
