# Supplementary material for: Long‐term cell fate and functional maintenance of human hepatocyte through stepwise culture configuration
Source: FASEB J. 2023 Jan 6;37(2):e22750. doi: 10.1096/fj.202201292RR (PMC9830592; doi:10.1096/fj.202201292RR)
Supplement: Supplementary file 1 — Figure S1. [file FSB2-37-0-s008.pptx]

## Slide 1
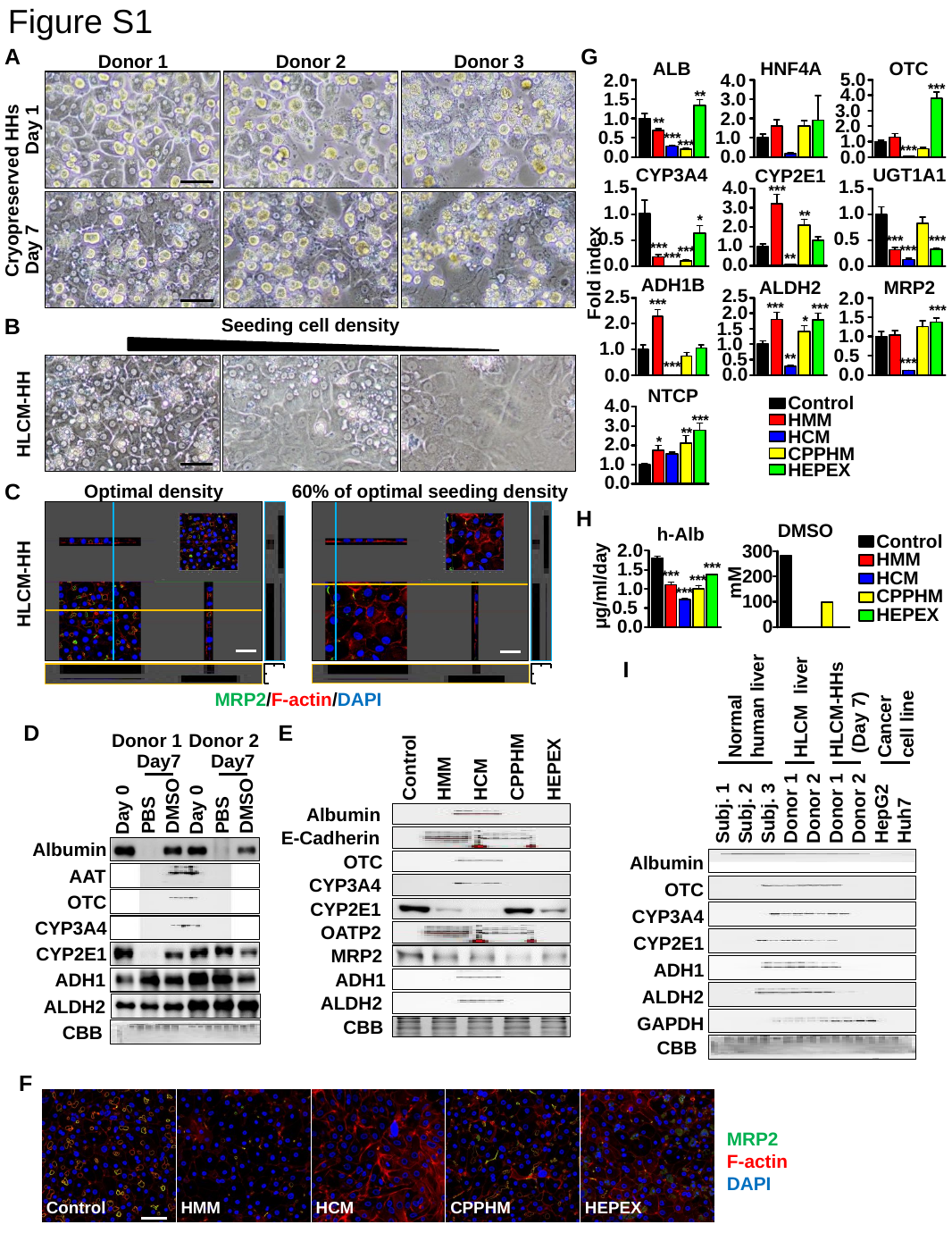

Figure S1
A
G
ALB
2.0
**
1.5
1.0
**
0.5
***
***
0.0
HNF4A
4.0
3.0
2.0
1.0
0.0
OTC
5.0
4.0
3.0
2.0
1.0
0.0
***
***
CYP3A4
1.5
1.0
0.5
0.0
*
***
***
***
UGT1A1
1.5
1.0
0.5
0.0
***
***
***
CYP2E1
4.0
***
3.0
**
2.0
1.0
**
0.0
ADH1B
2.5
2.0
1.0
0.0
***
***
ALDH2
2.5
2.0
1.5
1.0
0.5
0.0
***
***
*
**
MRP2
2.0
1.5
1.0
0.5
0.0
***
***
NTCP
4.0
3.0
2.0
1.0
0.0
***
**
*
Donor 1
Donor 2
Donor 3
Day 1
Day 7
Cryopreserved HHs
Fold index
B
Seeding cell density
HLCM-HH
Control
HMM
HCM
CPPHM
HEPEX
C
Optimal density
60% of optimal seeding density
HLCM-HH
MRP2/F-actin/DAPI
H
DMSO
h-Alb
Control
2.0
1.5
1.0
0.5
0.0
300
200
100
0
HMM
***
***
***
***
HCM
mM
μg/ml/day
CPPHM
HEPEX
Normal human liver
HLCM liver
HLCM-HHs (Day 7)
I
Cancer cell line
HEPEX
CPPHM
Control
HCM
HMM
Albumin
E-Cadherin
OTC
CYP3A4
CYP2E1
OATP2
MRP2
ADH1
ALDH2
CBB
D
E
Donor 1
Donor 2
Day7
Day7
Donor 1
Donor 1
PBS
PBS
DMSO
Donor 2
Huh7
HepG2
Subj. 2
DMSO
Subj. 3
Donor 2
Day 0
Day 0
Subj. 1
Albumin
Albumin
AAT
OTC
OTC
CYP3A4
CYP3A4
CYP2E1
CYP2E1
ADH1
ADH1
ALDH2
ALDH2
GAPDH
CBB
CBB
F
Control
HMM
HCM
CPPHM
HEPEX
MRP2
F-actin
DAPI

## Slide 2
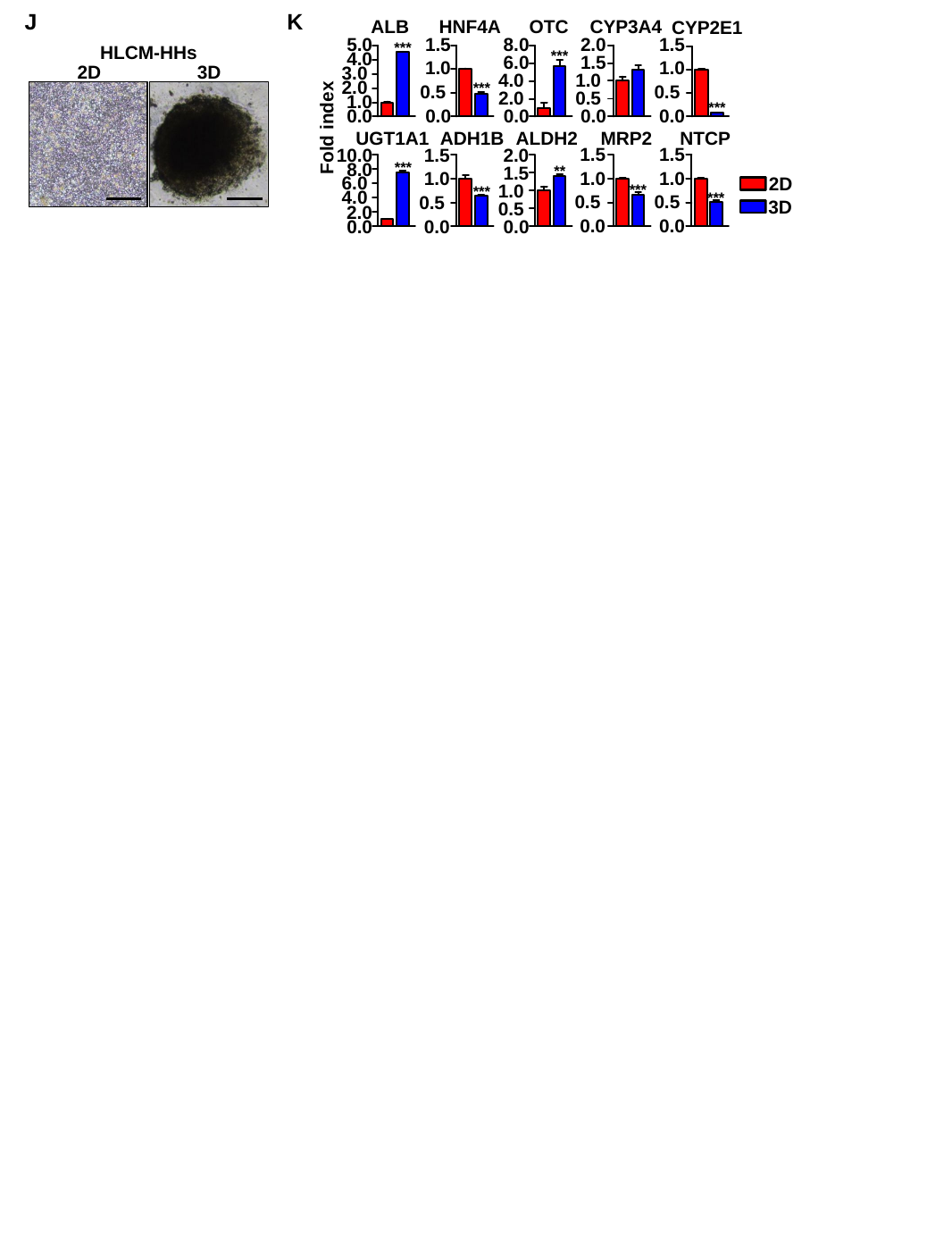

J
K
ALB
5.0
4.0
3.0
2.0
1.0
0.0
***
HNF4A
1.5
1.0
0.5
0.0
***
OTC
8.0
6.0
4.0
2.0
0.0
***
CYP3A4
2.0
1.5
1.0
0.5
0.0
CYP2E1
1.5
1.0
0.5
0.0
***
Fold index
MRP2
1.5
1.0
0.5
0.0
***
NTCP
1.5
1.0
0.5
0.0
***
UGT1A1
10.0
8.0
6.0
4.0
2.0
0.0
***
ADH1B
1.5
1.0
0.5
0.0
***
ALDH2
2.0
1.5
1.0
0.5
0.0
**
2D
3D
HLCM-HHs
3D
2D
