# Supplementary material for: Long‐term cell fate and functional maintenance of human hepatocyte through stepwise culture configuration
Source: FASEB J. 2023 Jan 6;37(2):e22750. doi: 10.1096/fj.202201292RR (PMC9830592; doi:10.1096/fj.202201292RR)
Supplement: Supplementary file 2 — Figure S2. [file FSB2-37-0-s004.pptx]

## Slide 1
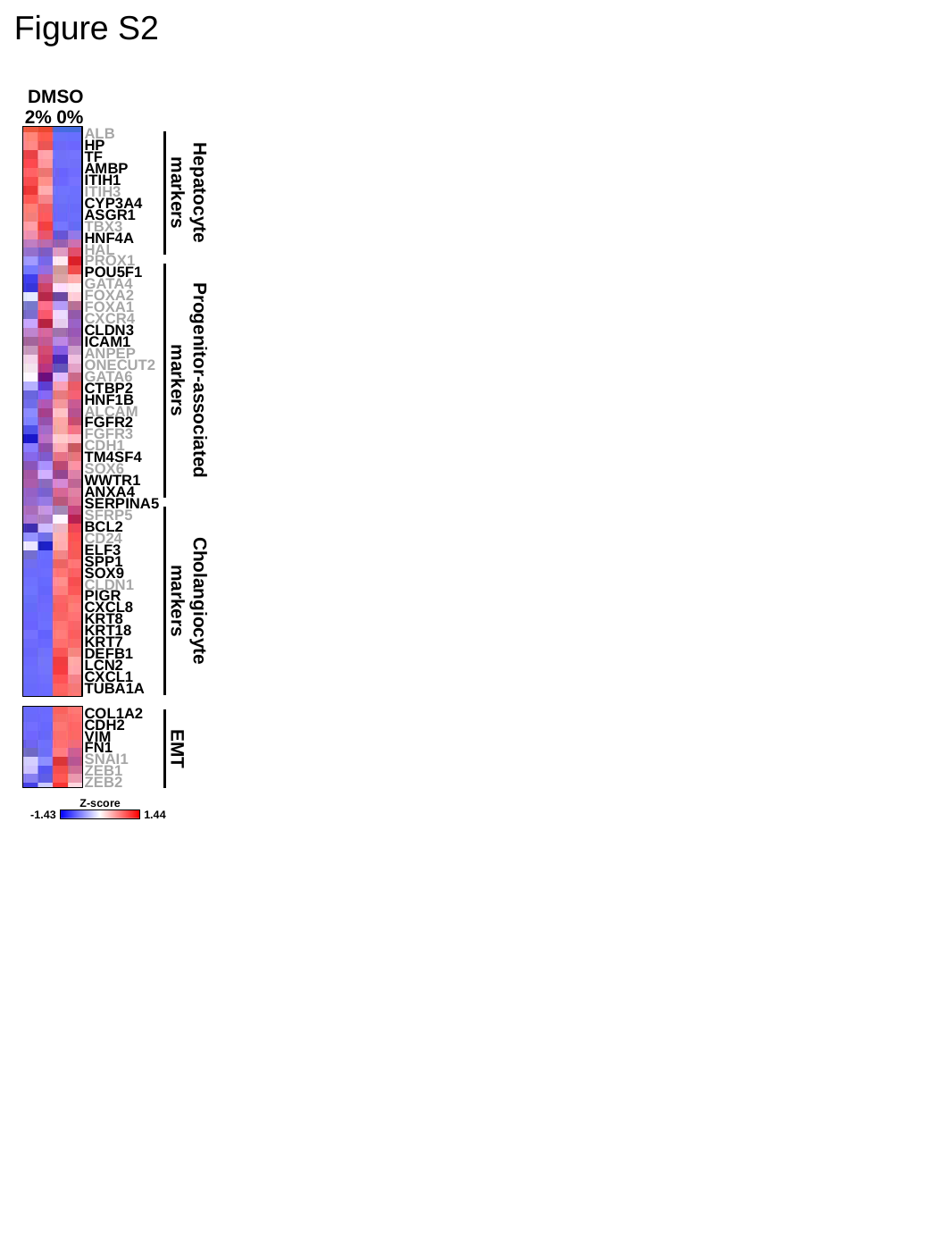

Figure S2
DMSO
Hepatocyte
markers
Progenitor-associated
markers
Cholangiocyte
markers
EMT
ALB
HP
TF
AMBP
ITIH1
ITIH3
CYP3A4
ASGR1
TBX3
HNF4A
HAL
PROX1
POU5F1
GATA4
FOXA2
FOXA1
CXCR4
CLDN3
ICAM1
ANPEP
ONECUT2
GATA6
CTBP2
HNF1B
ALCAM
FGFR2
FGFR3
CDH1
TM4SF4
SOX6
WWTR1
ANXA4
SERPINA5
SFRP5
BCL2
CD24
ELF3
SPP1
SOX9
CLDN1
PIGR
CXCL8
KRT8
KRT18
KRT7
DEFB1
LCN2
CXCL1
TUBA1A
COL1A2
CDH2
VIM
FN1
SNAI1
ZEB1
ZEB2
Z-score
-1.43
1.44
2%
0%
