# Supplementary material for: Long‐term cell fate and functional maintenance of human hepatocyte through stepwise culture configuration
Source: FASEB J. 2023 Jan 6;37(2):e22750. doi: 10.1096/fj.202201292RR (PMC9830592; doi:10.1096/fj.202201292RR)
Supplement: Supplementary file 3 — Figure S3. [file FSB2-37-0-s005.pptx]

## Slide 1
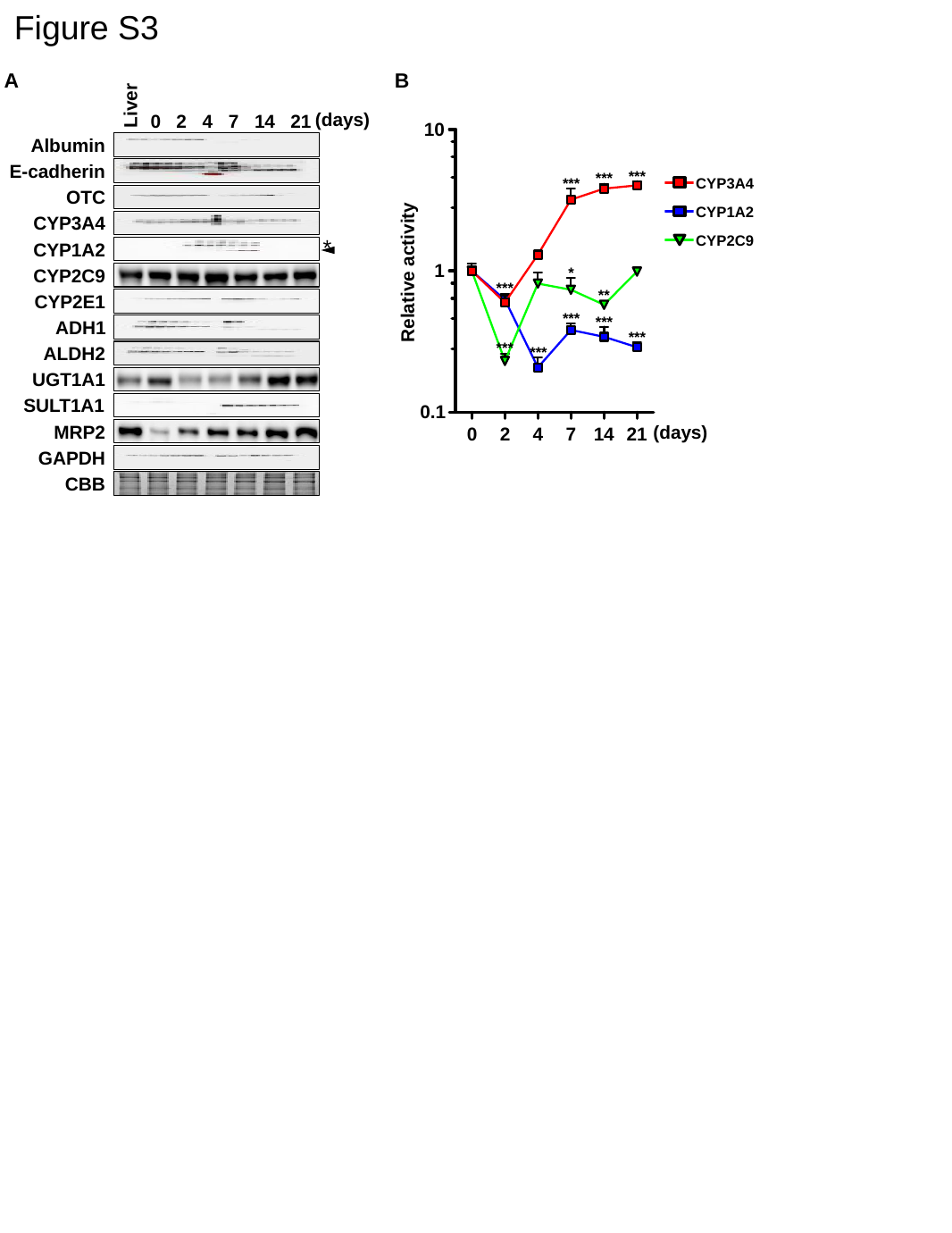

Figure S3
A
B
Liver
(days)
0
2
4
7
14
21
10
1
0.1
Relative activity
***
***
***
*
***
**
***
***
***
***
***
CYP3A4
CYP1A2
CYP2C9
(days)
0
2
4
7
14
21
Albumin
E-cadherin
OTC
CYP3A4
*
CYP1A2
CYP2C9
CYP2E1
ADH1
ALDH2
UGT1A1
SULT1A1
MRP2
GAPDH
CBB
