# Supplementary material for: Long‐term cell fate and functional maintenance of human hepatocyte through stepwise culture configuration
Source: FASEB J. 2023 Jan 6;37(2):e22750. doi: 10.1096/fj.202201292RR (PMC9830592; doi:10.1096/fj.202201292RR)
Supplement: Supplementary file 4 — Figure S4. [file FSB2-37-0-s010.pptx]

## Slide 1
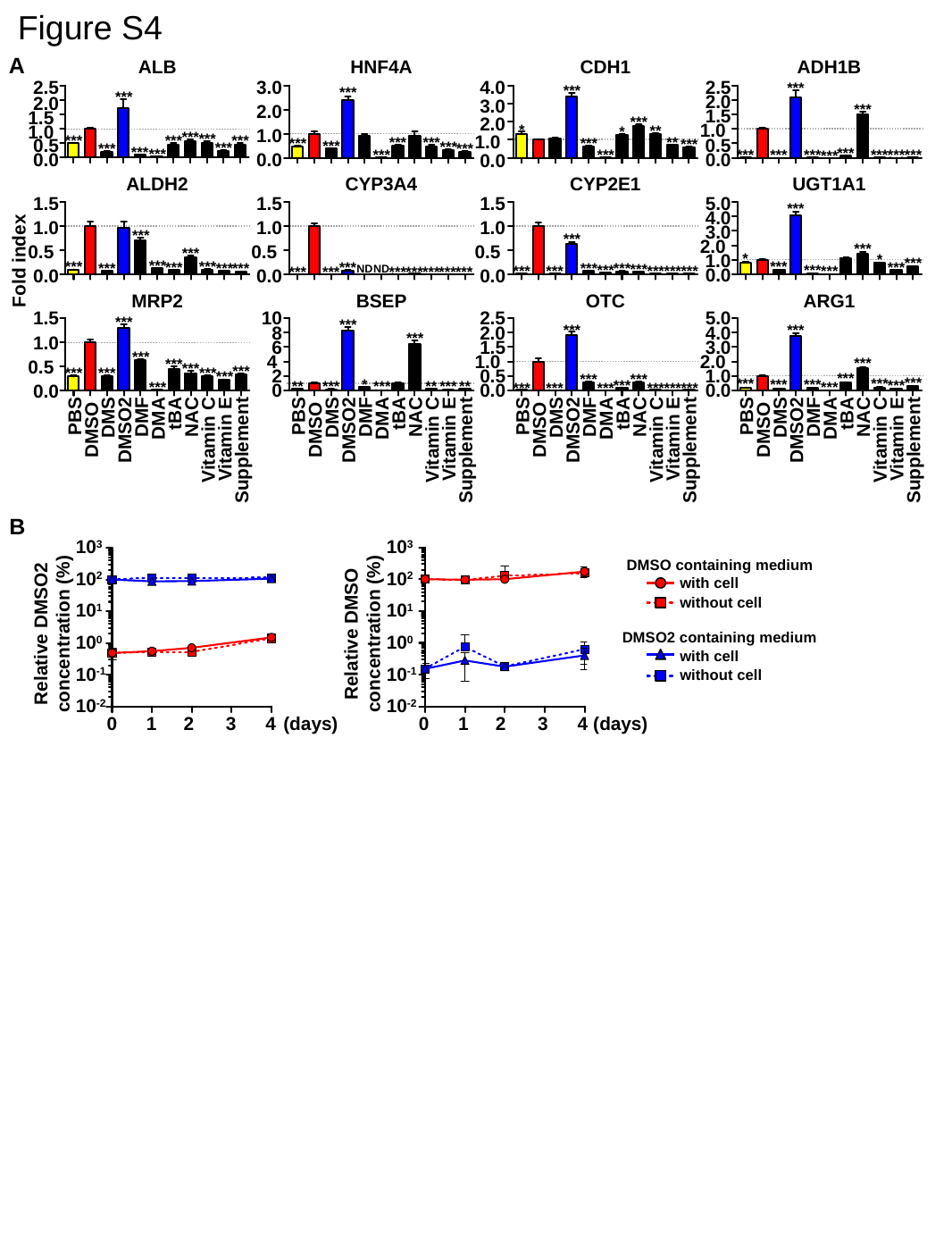

Figure S4
A
ALB
HNF4A
CDH1
ADH1B
2.5
2.0
1.5
1.0
0.5
0.0
3.0
2.0
1.0
0.0
4.0
3.0
2.0
1.0
0.0
2.5
2.0
1.5
1.0
0.5
0.0
***
***
***
***
***
***
***
***
***
***
***
***
*
**
**
***
***
***
*
***
***
***
***
***
***
***
***
***
***
***
***
***
***
***
***
***
***
ALDH2
CYP3A4
CYP2E1
UGT1A1
1.5
1.0
0.5
0.0
1.5
1.0
0.5
0.0
1.5
1.0
0.5
0.0
5.0
4.0
3.0
2.0
1.0
0.0
***
***
*
*
***
***
***
***
***
***
***
***
***
***
***
***
***
***
***
***
***
***
***
***
***
***
***
***
Fold index
***
***
***
***
***
***
***
***
ND
ND
MRP2
BSEP
OTC
ARG1
1.5
1.0
0.5
0.0
10
8
6
4
2
0
2.5
2.0
1.5
1.0
0.5
0.0
5.0
4.0
3.0
2.0
1.0
0.0
***
***
***
***
***
***
***
***
***
***
***
***
*
**
**
**
***
***
***
***
***
***
***
***
***
***
***
***
***
***
***
***
***
***
***
***
***
***
***
DMS
DMF
tBA
DMSO
DMSO2
DMA
NAC
PBS
Vitamin E
Vitamin C
Supplement
DMS
DMF
tBA
DMSO
DMSO2
DMA
NAC
PBS
Vitamin E
Vitamin C
Supplement
DMS
DMF
tBA
DMSO
DMSO2
DMA
NAC
PBS
Vitamin E
Vitamin C
Supplement
DMS
DMF
tBA
DMSO
DMSO2
DMA
NAC
PBS
Vitamin E
Vitamin C
Supplement
B
103
102
101
100
10-1
10-2
103
102
101
100
10-1
10-2
DMSO containing medium
with cell
without cell
DMSO2 containing medium
with cell
without cell
Relative DMSO2 concentration (%)
Relative DMSO concentration (%)
(days)
(days)
0
1
2
3
4
0
1
2
3
4
