# Supplementary material for: Long‐term cell fate and functional maintenance of human hepatocyte through stepwise culture configuration
Source: FASEB J. 2023 Jan 6;37(2):e22750. doi: 10.1096/fj.202201292RR (PMC9830592; doi:10.1096/fj.202201292RR)
Supplement: Supplementary file 5 — Figure S5. [file FSB2-37-0-s012.pptx]

## Slide 1
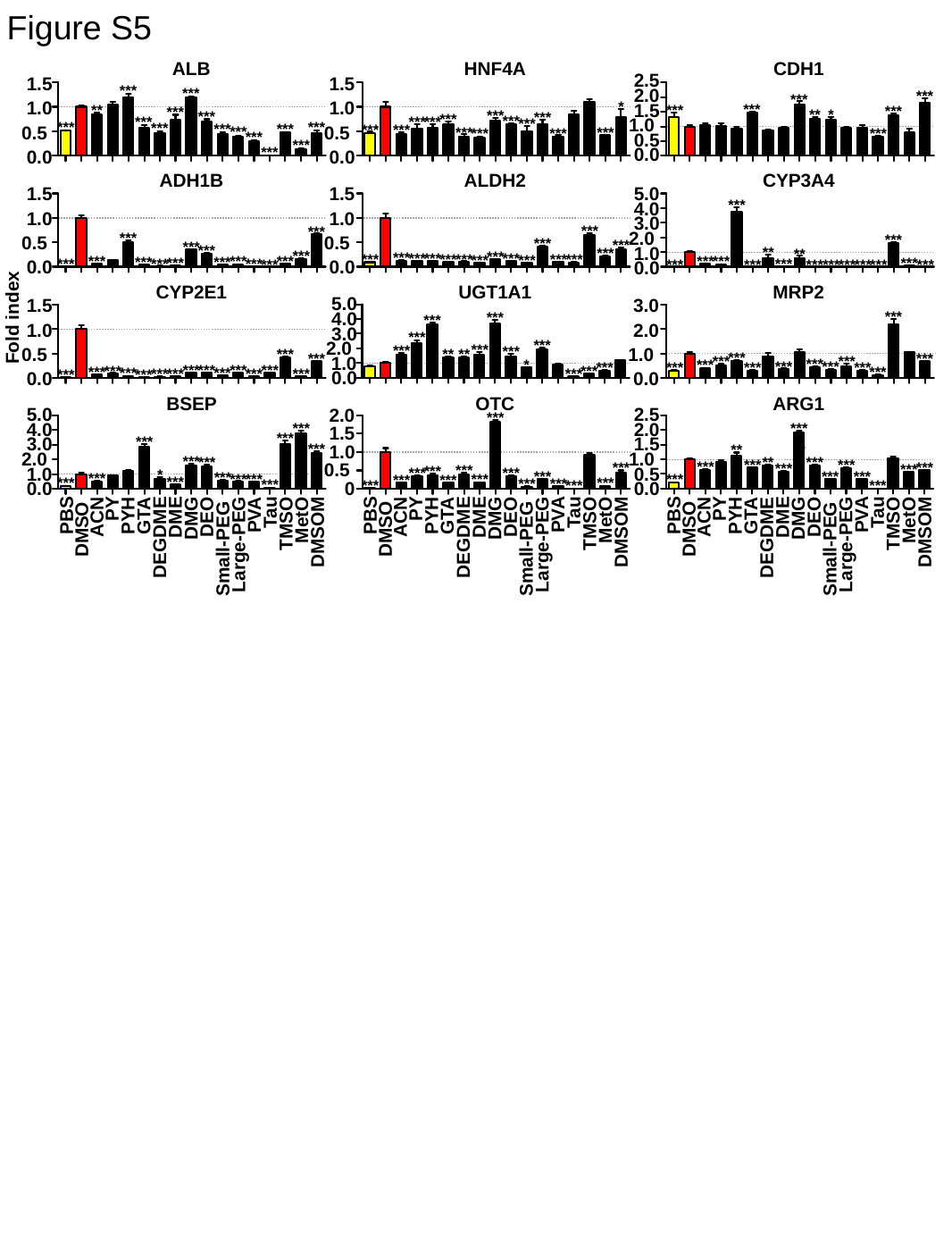

Figure S5
ALB
HNF4A
CDH1
2.5
2.0
1.5
1.0
0.5
0.0
1.5
1.0
0.5
0.0
1.5
1.0
0.5
0.0
ADH1B
ALDH2
CYP3A4
1.5
1.0
0.5
0.0
1.5
1.0
0.5
0.0
5.0
4.0
3.0
2.0
1.0
0.0
CYP2E1
UGT1A1
MRP2
5.0
4.0
3.0
2.0
1.0
0.0
1.5
1.0
0.5
0.0
3.0
2.0
1.0
0.0
BSEP
OTC
ARG1
5.0
4.0
3.0
2.0
1.0
0.0
2.5
2.0
1.5
1.0
0.5
0.0
2.0
1.5
1.0
0.5
0
PBS
DEO
ACN
PYH
Tau
MetO
DMSO
PY
GTA
TMSO
DMSOM
DMG
DEGDME
DME
Small-PEG
PVA
Large-PEG
PBS
DEO
ACN
PYH
Tau
MetO
DMSO
PY
GTA
TMSO
DMSOM
DMG
DEGDME
DME
Small-PEG
PVA
Large-PEG
PBS
DEO
ACN
PYH
Tau
MetO
DMSO
PY
GTA
TMSO
DMSOM
DMG
DEGDME
DME
Small-PEG
PVA
Large-PEG
Fold index
***
***
**
***
***
***
***
***
***
***
***
***
***
***
***
***
***
***
***
***
**
*
***
*
***
***
***
***
***
***
***
***
***
***
***
***
***
***
***
**
**
***
***
***
***
***
***
***
***
***
***
***
***
***
***
***
***
***
***
***
***
***
***
***
***
***
***
***
***
***
***
***
***
***
***
***
***
***
***
***
***
***
***
***
***
***
***
***
***
***
***
***
***
***
***
***
***
***
***
***
***
***
***
***
**
**
*
***
***
***
***
***
***
***
***
***
***
***
***
***
***
***
***
***
***
***
***
***
***
***
***
***
***
***
***
***
***
***
***
***
***
***
***
***
***
***
***
*
***
***
***
***
***
***
***
***
**
**
***
**
***
***
***
***
***
***
***
***
***
***
