# Supplementary material for: Long‐term cell fate and functional maintenance of human hepatocyte through stepwise culture configuration
Source: FASEB J. 2023 Jan 6;37(2):e22750. doi: 10.1096/fj.202201292RR (PMC9830592; doi:10.1096/fj.202201292RR)
Supplement: Supplementary file 6 — Figure S6. [file FSB2-37-0-s003.pptx]

## Slide 1
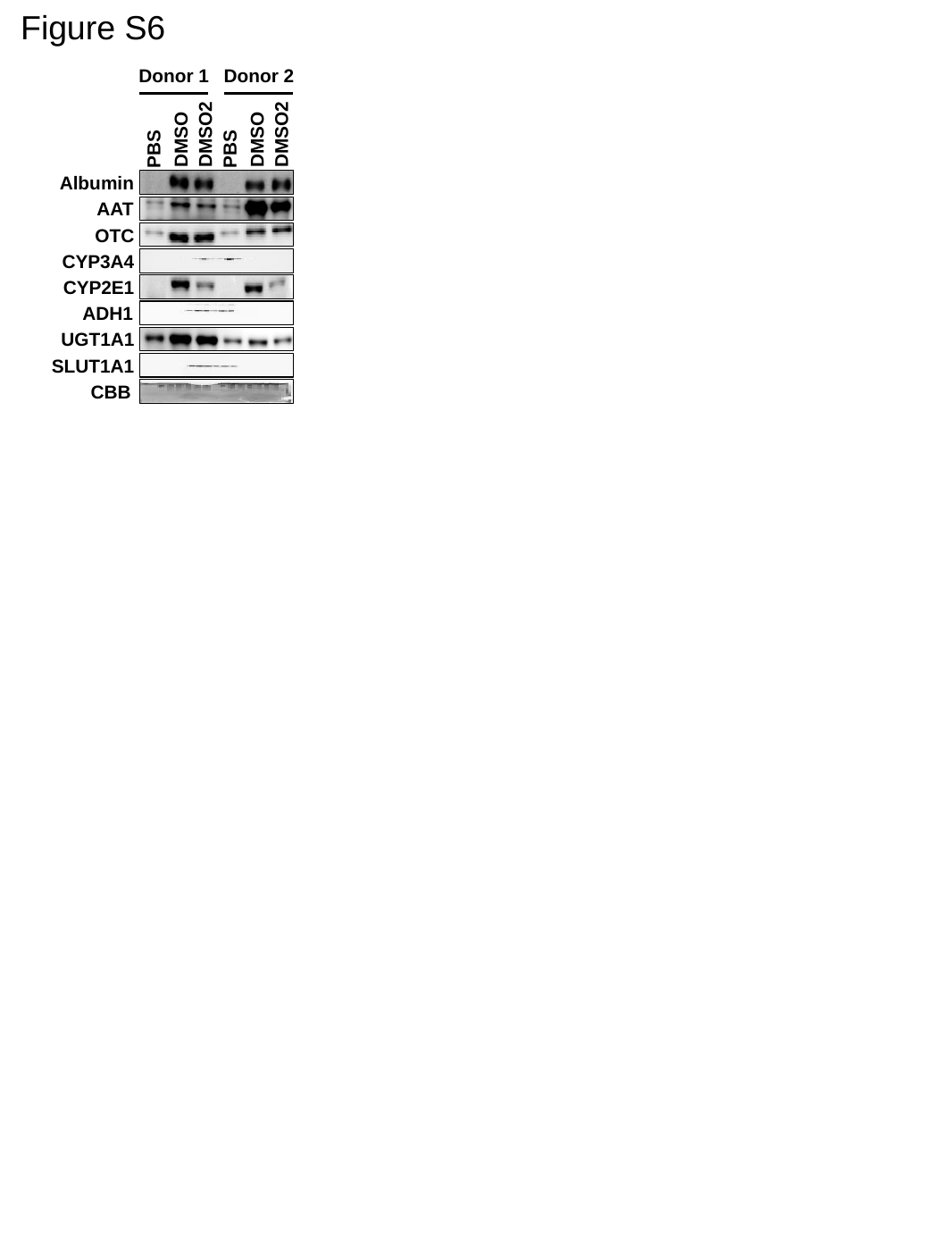

Figure S6
Donor 1
Donor 2
DMSO2
PBS
DMSO
PBS
DMSO2
DMSO
Albumin
AAT
OTC
CYP3A4
CYP2E1
ADH1
UGT1A1
SLUT1A1
CBB
