# Supplementary material for: Long‐term cell fate and functional maintenance of human hepatocyte through stepwise culture configuration
Source: FASEB J. 2023 Jan 6;37(2):e22750. doi: 10.1096/fj.202201292RR (PMC9830592; doi:10.1096/fj.202201292RR)
Supplement: Supplementary file 7 — Figure S7. [file FSB2-37-0-s006.pptx]

## Slide 1
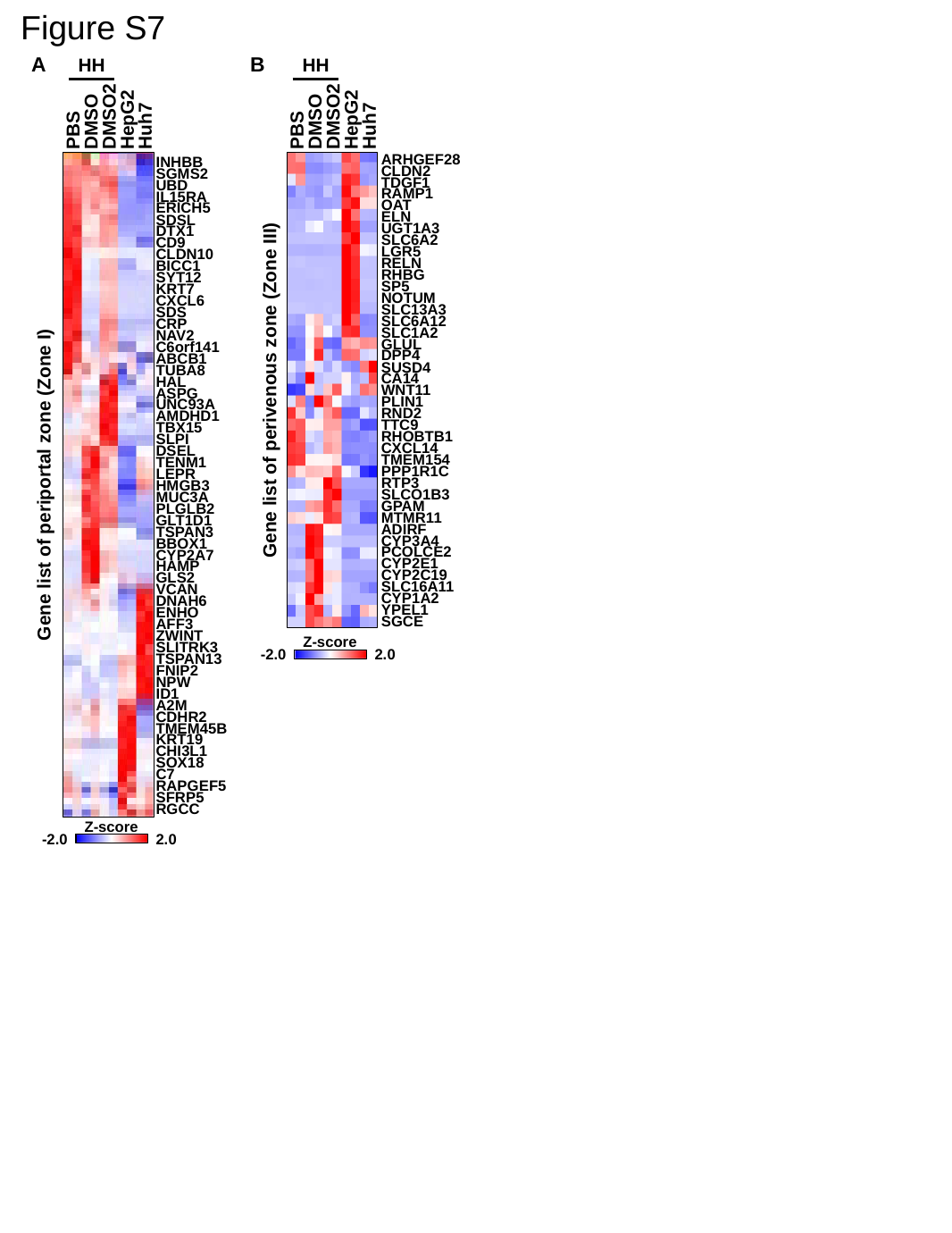

Figure S7
A
B
HH
DMSO2
HepG2
DMSO
Huh7
PBS
HH
DMSO2
HepG2
DMSO
Huh7
PBS
ARHGEF28
CLDN2
TDGF1
RAMP1
OAT
ELN
UGT1A3
SLC6A2
LGR5
RELN
RHBG
SP5
NOTUM
SLC13A3
SLC6A12
SLC1A2
GLUL
DPP4
SUSD4
CA14
WNT11
PLIN1
RND2
TTC9
RHOBTB1
CXCL14
TMEM154
PPP1R1C
RTP3
SLCO1B3
GPAM
MTMR11
ADIRF
CYP3A4
PCOLCE2
CYP2E1
CYP2C19
SLC16A11
CYP1A2
YPEL1
SGCE
INHBB
SGMS2
UBD
IL15RA
ERICH5
SDSL
DTX1
CD9
CLDN10
BICC1
SYT12
KRT7
CXCL6
SDS
CRP
NAV2
C6orf141
ABCB1
TUBA8
HAL
ASPG
UNC93A
AMDHD1
TBX15
SLPI
DSEL
TENM1
LEPR
HMGB3
MUC3A
PLGLB2
GLT1D1
TSPAN3
BBOX1
CYP2A7
HAMP
GLS2
VCAN
DNAH6
ENHO
AFF3
ZWINT
SLITRK3
TSPAN13
FNIP2
NPW
ID1
A2M
CDHR2
TMEM45B
KRT19
CHI3L1
SOX18
C7
RAPGEF5
SFRP5
RGCC
Gene list of perivenous zone (Zone III)
Gene list of periportal zone (Zone I)
Z-score
-2.0
2.0
Z-score
-2.0
2.0
