# Supplementary material for: Long‐term cell fate and functional maintenance of human hepatocyte through stepwise culture configuration
Source: FASEB J. 2023 Jan 6;37(2):e22750. doi: 10.1096/fj.202201292RR (PMC9830592; doi:10.1096/fj.202201292RR)
Supplement: Supplementary file 8 — Table S1. [file FSB2-37-0-s011.docx]

| **Table S1. List of chemical and product for cell culture and treatment** | | | | |
| --- | --- | --- | --- | --- |
| **Chemicals or products name** | **Abbreviations** | **Manufactures** | **Cat No.** | **Working conc. or dilution** |
| HMM™ Basal Medium and SingleQuots™ Kit | HMM | Lonza | CC-3197, CC-4192 | - |
| HCMTM Hepatocyte Culture Medium BulletKit^TM^ | HCM | Lonza | CC-3198 | - |
| HepExtend™ Supplement (50X) | HEPEX | Thermo Scientific | A2737501 | - |
| Cellartis® Power™ Primary HEP Medium | CPPHM | Takara | Y20020 | - |
| dimethyl sulfoxide | DMSO | MilliporeSigma | D2650 | 281.6mM |
| dimethyl sulfide | DMS | MilliporeSigma | 471577 | 281.6mM |
| dimethyl sulfone | DMSO2 | MilliporeSigma | M81705 | 140.8mM |
| N,N-dimethylformamide | DMF | MilliporeSigma | D4551 | 281.6mM |
| dimethylacetamide | DMA | MilliporeSigma | 271012 | 70.4mM |
| tert-Butanol | tBA | MilliporeSigma | 471712 | 281.6mM |
| N-acetyl-L-cysteine | NAC | MilliporeSigma | A9165 | 10mM |
| L-ascorbic acid | Vitamin C | MilliporeSigma | A8960 | 10mM |
| α-tocopherol acetate | Vitamin E | MilliporeSigma | T1157 | 1mM |
| Antioxidant Supplement | Supplement | MilliporeSigma | A1345 | x1000 dilution |
| acetonitrile | ACN | MilliporeSigma | 271004 | 281.6mM |
| pyridine | PY | MilliporeSigma | 270970 | 56.32mM |
| pyridine hydrochloride | PYH | MilliporeSigma | 307475 | 28.16mM |
| glyceryl triacetate | GTA | MilliporeSigma | 90240 | 5.632mM |
| diethylene glycol dimethyl ether | DEGDME | MilliporeSigma | 04143 | 56.32mM |
| 1,2-dimethoxyethane | DME | MilliporeSigma | 259527 | 281.6mM |
| diethylene glycol | DEG | MilliporeSigma | 93171 | 140.8mM |
| 1,4-dioxane | DEO | MilliporeSigma | 296309 | 281.6mM |
| poly(ethylene glycol) large PEG 20000 | Small-PEG | MilliporeSigma | 95172 | 2.5mM |
| poly(ethylene glycol) small PEG 400 | Large-PEG | MilliporeSigma | 91893 | 62.5mM |
| poly(vinyl alcohol) | PVA | MilliporeSigma | P8136 | 0.5mM |
| taurine | Tau | MilliporeSigma | T0625 | 140.8mM |
| tetramethylene sulfoxide | TMSO | Alfa Aesar | A17502 | 140.8mM |
| L-methionine sulfoxide | MetO | Alfa Aesar | J62873-03 | 281.6mM |
| dimethylsulfoximine | DMSOM | TCI America | D4971 | 281.6mM |
| rifampicin | - | MilliporeSigma | R7382 | 0.5 - 50 µM |
| carbamazepine | - | MilliporeSigma | C4024 | 0.5 - 50 µM |
| ethanol | - | MilliporeSigma | 1.00986 | 10 - 50 mM |
| isoniazid | - | MilliporeSigma | I3377 | 0.01 - 1 mM |
