# Supplementary material for: Long‐term cell fate and functional maintenance of human hepatocyte through stepwise culture configuration
Source: FASEB J. 2023 Jan 6;37(2):e22750. doi: 10.1096/fj.202201292RR (PMC9830592; doi:10.1096/fj.202201292RR)
Supplement: Supplementary file 9 — Table S2. [file FSB2-37-0-s009.docx]

| **Table S2. List of antibodies used for immunoblotting and immunofluorescence** | | | |  |  |  |
| --- | --- | --- | --- | --- | --- | --- |
| **Primary antibodies** | **Obtained from** | **Mono/Poly Clonal** | **Cat No.** | **Manufactures** | **Dilution** | **Application** |
| Albumin | Rabbit | Monoclonal (EPSISR1) | ab137885 | Abcam | 1:1000 | WB |
| alpha 1 Antitrypsin (AAT) | Rabbit | Polyclonal | GTX112707 | Genetex | 1:1000 | WB |
| E-Cadherin | Rabbit | Polyclonal | GTX100443 | Genetex | 1:1000 | WB |
| OTC | Rabbit | Polyclonal | GTX105140 | Genetex | 1:1000 | WB |
| CYP3A4 | Mouse | Monoclonal (clone:3H8) | GTX60577 | Genetex | 1:1000 | WB |
| CYP2C9 | Rabbit | Polyclonal | A6219 | Abclonal | 1:1000 | WB |
| CYP2E1 | Rabbit | Polyclonal | ab28146 | Abcam | 1:1000 | WB |
| OATP2 | Rabbit | Polyclonal | GTX31621 | Genetex | 1:1000 | WB |
| NTPC | Rabbit | Polyclonal | GTX17693 | Genetex | 1:1000 | WB |
| MRP2 | Mouse | Monoclonal (M2III-6) | GTX23373 | Genetex | 1:100 | IF |
| MRP2 | Rabbit | Polyclonal | GTX54899 | Genetex | 1:1000 | WB |
| ADH | Rabbit | Monoclonal (EPR4440) | ab108197 | Abcam | 1:1000 | WB |
| ALDH2 | Rabbit | Monoclonal (EPR4493) | ab108306 | Abcam | 1:1000 | WB |
| UGT1A1 | Rabbit | Polyclonal | 23495-1-AP | Proteintech | 1:1000 | WB |
| UGT2B7 | Rabbit | Polyclonal | 16661-1-AP | Proteintech | 1:1000 | WB |
| SULT1A1 | Rabbit | Polyclonal | 10911-2-AP | Proteintech | 1:1000 | WB |
| β-Actin | Mouse | Monoclonal (AC-15) | sc-69879 | Santa Cruz | 1:1000 | WB |
| GAPDH | Mouse | Monoclonal (mAbcam 9484) | ab9484 | Abcam | 1:1000 | WB |
