# Supplementary material for: Long‐term cell fate and functional maintenance of human hepatocyte through stepwise culture configuration
Source: FASEB J. 2023 Jan 6;37(2):e22750. doi: 10.1096/fj.202201292RR (PMC9830592; doi:10.1096/fj.202201292RR)
Supplement: Supplementary file 10 — Table S3. [file FSB2-37-0-s001.docx]

| **Table S3. Primer sequence used for RT-PCR** | | |
| --- | --- | --- |
| **Gene name** | **Forward (5' - 3')** | **Reverse (5' - 3')** |
| ADH1B | CCCGGAGAGCAACTACTGC | AACCAGTCGAGAATCCACAGC |
| ALDH2 | CCAGCTCCAAGGTCACTCTCTTG | TGAGGATGTGGACAAAGTGG |
| ALB | GGATGAAGGGAAGGCTTCGT | TGGGAAATCTCTGGCTCAGG |
| ARG1 | TGGACAGACTAGGAATTGGCA | CCAGTCCGTCAACATCAAAACT |
| BSEP | AAATATGCTTTTGGGTCATTG | GTCAGCTATGGCATCATTG |
| CDH1 | CGAGAGCTACACGTTCACGG | GGGTGTCGAGGGAAAAATAGG |
| CYP2C9 | CCAGATCTGCAATAATTTTTCTC | CAAGCTTTCAATAGTAAATTCAGATG |
| CYP2E1 | CGAGAGCTACACGTTCACGG | GGGTGTCGAGGGAAAAATAGG |
| CYP3A4 | ACTGCCTTTTTTGGGAAATA | GGCTGTTGACCATCATAAAAG |
| GAPDH | GGAGTCAACGGATTTGGT | AAGATGGTGATGGGATTTCCA |
| HNF4A | GACCGCCAGTATGACTCG | CGTTGGTTCCCATATGTTCC |
| MRP2 | TCCAACTGTGCTTCAAGC | GGCATCCACAGACATCAG |
| NTCP | AAGGACAAGGTGCCCTATAAAGG | TTGAGGACGATCCCTATGGTG |
| OTC | CGGCCCGTGTATTGTCTAGC | TAGCCAGGGTGTCCAAATCTG |
| UGT1A1 | TTGATCCCAGTGGATGGC | ATGCTCCGTCTCTGATGTACAAC |
