# Supplementary material for: Long‐term cell fate and functional maintenance of human hepatocyte through stepwise culture configuration
Source: FASEB J. 2023 Jan 6;37(2):e22750. doi: 10.1096/fj.202201292RR (PMC9830592; doi:10.1096/fj.202201292RR)
Supplement: Supplementary file 11 — Table S4. [file FSB2-37-0-s007.docx]

| **Table S4. The viability and platability of cryopreserved PHH and HLCM-HH.** | | | |
| --- | --- | --- | --- |
|  |  | **Viability (%)** | **Platability (%)** |
| **PHH** | **Donor 1** | 91.28 | 50.38 ± 0.20 |
|  | **Donor 2** | 92.48 | 31.76 ± 4.08 |
|  | **Donor 3** | 94.53 | 19.78 ± 2.65 |
| **HLCM-HH** | **Animal 1** | 92.40 | 63.23 ± 3.51 |
|  | **Animal 2** | 94.06 | 71.32 ± 1.22 |
|  | **Animal 3** | 90.15 | 68.14 ± 4.63 |
|  | **Animal 4** | 89.36 | 56.3 ± 1.33 |
|  | **Animal 5** | 95.41 | 60.92 ± 3.78 |
