# Supplementary material for: Long‐term cell fate and functional maintenance of human hepatocyte through stepwise culture configuration
Source: FASEB J. 2023 Jan 6;37(2):e22750. doi: 10.1096/fj.202201292RR (PMC9830592; doi:10.1096/fj.202201292RR)
Supplement: Supplementary file 12 — Appendix S1. Supporting Information. [file FSB2-37-0-s002.docx]

**Supplemental Figure Legend**

**Figure S1. A.** Bright-field microscopic analysis of cryopreserved PHHs (three different donors) seeded at the optimal cell density followed by incubation with 2% DMSO containing medium for 1 (top) or 7 days (bottom). **B.** Bright-field microscopic analysis of HLCM-HHs plated at decreasing seeding density (100, 80, and 60% of the optimal density) were cultured for 7 days with 2% DMSO containing medium. Bar =50 µm. **C.** IFA of HLCM-HHs plated at indicated seeding density followed by incubation with 2% DMSO containing medium for 7 days. The side view analysis of the merged z-stack image (view in the x and y plane indicated as the orange and blue line, respectively. MRP2 (Green), phalloidin (F-action: Red) and DAPI (Blue) Bar =25 μm. **D**. IB analysis of indicated hepatocyte marker gene expression in cryopreserved PHHs from two different donors that were cultured in the absence (PBS) or presence of 2% DMSO for 7 days. **E-G.** Freshly isolated HLCM-HHs were cultured with medium containing 2% DMSO (control) or commercially available hepatocyte culture medium (Hepatocyte Basal Medium; HMM^TM^, Hepatocyte Culture Medium; HCM^TM^, Cellartis® Power™ Primary HEP Medium; CPPHM, HepExtend™ Medium (HEPEX)) for 7 days, which were then subjected to the evaluation of hepatocyte marker gene expression at the level of protein (E) via IB analysis, the morphological appearance via IFA (F) and marker genes expression at the level of transcript (G) via RT-qPCR, respectively. RT-qPCR results represent the relative fold index to the average of the control condition (2% DMSO containing medium) normalized by the value of GAPDH. For the IB analysis, an equal amount of protein samples from each condition were applied, with the CBB staining of the SDS-PAGE gel serving as a loading control. MRP2 (Green), phalloidin (F-action: Red) and DAPI (Blue). Bar =25 μm. Graph bars represent mean ± SD; *, ** and *** indicate *p* < 0.05, *p* < 0.01, and *p* < 0.001, versus control value by one‐way ANOVA. The culture supernatants were subjected to the assessment of albumin secretion (H; left) and DMSO concentration (H; right) via HPLC-MS. **I**. IB analysis of indicated hepatocyte marker gene expression in normal human liver tissue, the liver tissue of HLCM harboring human hepatocytes from two different, HLCM-HHs isolated from these HLCM livers, and hepatoma cell lines (HepG2 and Huh7 cells) cultured for 7 days with 2% DMSO-supplemented culture medium. **J-K.** Freshly isolated HLCM-HH either plated into the type I collagen coated cell culture dish (2D; left) or seeded into 96-well format Ultra-Low Attachment Surface Plate (3D; right) in the presence of 2% DMSO-containing medium for 7 days, which were then subjected to bright-field microscopic analysis (H) and the assessment of hepatocyte marker gene expression via RT-qPCR (I). Bar =200 µm. RT-qPCR results are shown as the relative fold index to the average of the 2D cultured samples normalized by the value of GAPDH. Graph bars represent mean ± SD; ** and *** indicate *p* < 0.01 and *p* < 0.001, versus 2D value by Student’s *t*-tests.

**Figure S2.** Fleshly isolated HLCM-HHs cultured with 2% DMSO-containing medium for 7 days were further maintained either in the absence or presence of 2% DMSO for 7 days, which were then subjected to mRNA-sequencing analysis. Heat map analysis demonstrates the comparison of normalized transcriptome data for selected gene sets representative of terminally differentiated hepatocytes (hepatocyte markers), hepatic progenitor cells (progenitor-associated markers), cholangiocytes (cholangiocytes markers), and genes known to be upregulated during the process of epithelial–mesenchymal transition (EMT). Gene with FDR <0.1 and a fold change of less or greater than 2 are shown in black letters.

**Figure S3. A-B.** Freshly isolated HLCM-HHs seeded at the optimal cell density were incubated with medium containing 2% DMSO for indicated duration followed by the assessment of hepatocyte marker gene expression via IB (A) analysis as well as enzymatic activities of CYP3A4, CYP1A2, and CYP2C9 (B). The liver tissue of HLCM was included in the analysis as an additional control. The CBB staining of the SDS-PAGE gel serves as the loading control. * and arrowhead indicate nonspecific and specific signal, respectively (A). Graph symbols represent mean ± SD; ** and *** indicate p < 0.01*** and p < 0.001, versus Day 0 value by Student’s *t*-tests (B).

**Figure S4.** **A**. Freshly isolated HLCM-HHs were cultured with 2% DMSO containing media for 7 days were further maintained in the absence (PBS) or presence of indicated organic compound or for an additional 7 days, which were then subjected to the assessment of hepatocyte marker gene via RT-qPCR. The results are shown as the fold index relative to the average of the control (PBS) samples normalized by the value of GAPDH. Graph bars represent mean ± SD; *, ** and *** indicate *p* < 0.05, *p* < 0.01, and *p* < 0.001, versus DMSO value by one‐way ANOVA; ND., not detected. **B.** Media containing either 2% DMSO (281mM; red line) or DMSO2 (140mM; blue line) were incubated in the presence (solid line) or absence (dotted line) of HLCM-HHs that had completed the 7 days of recovery phase culture for up to 4 days followed by the measurement of DMSO (right) and DMSO2 (left) concentration via HPLC-MS. The graph demonstrates the concentration relative to the baseline.

**Figure S5.** Freshly isolated HLCM-HHs that had completed 7 days of the recovery phase culture with medium containing 2% DMSO were further maintained in the absence (PBS) or presence of indicated organic compound or for an additional 7 days, which were then subjected to the assessment of hepatocyte marker gene expression via RT-qPCR. The results are shown as the fold index relative to the average of the DMSO-treated samples normalized by the value of GAPDH. Graph bars represent mean ± SD; *, ** and *** indicate *p* < 0.05, *p* < 0.01, and *p* < 0.001, versus DMSO value by one‐way ANOVA.

**Figure S6.** Cryopreserved PHHs from two different donors that had completed 7 days of the recovery phase culture with 2% DMSO containing medium were further maintained in the absence (PBS) or presence of DMSO or DMSO2 for an additional 7 days, which were then subjected to the assessment of hepatocyte marker gene expression via IB. CBB staining of SDS-PAGE gel serves as the loading control.

**Figure S7. A-B.** Freshly isolated HLCM-HHs that had completed 7 days of the recovery phase culture in the presence of 2% DMSO were further maintained with either 2% DMSO or DMSO2 (140mM)-containing medium for an additional 7 days, which were then subjected to mRNA-sequencing analysis. HepG2 cells and Huh7 cells cultured with 2% DMSO containing medium for 7 days were included in the analysis as comparison groups. The normalized transcriptome data were applied for the assessment relative expression of genes known to be enriched in either zone I (A) or zone III (B)(McEnerney et al., 2017).

**Reference for Supplemental Figure Legend**

McEnerney, L., K. Duncan, B.R. Bang, S. Elmasry, M. Li, T. Miki, S.K. Ramakrishnan, Y.M. Shah, and T. Saito. 2017. Dual modulation of human hepatic zonation via canonical and non-canonical Wnt pathways. *Exp Mol Med*. 49:e413.
